# Intensive Cycloalkyl-Fused Pyridines for Aminopyridyl–Zinc–Heteroimidazoles Achieving High Efficiency toward the Ring-Opening Polymerization of Lactides

**DOI:** 10.3390/molecules29174150

**Published:** 2024-08-31

**Authors:** Yun Wang, Wenjuan Zhang, Pengjiang Zhu, Wei You, Xiaopan Xue, Rui Wang, Yanping Ma, Wen-Hua Sun

**Affiliations:** 1 Beijing Key Laboratory of Clothing Materials R&D and Assessment, Beijing Engineering Research Center of Textile Nanofiber, School of Materials Science and Engineering, Beijing Institute of Fashion Technology, Beijing 100029, China; 202020001129@stu.bift.edu.cn (Y.W.); zhupengjiang@yeah.net (P.Z.); 202120001113@stu.bift.edu.cn (X.X.); clywangrui@bift.edu.cn (R.W.); 2 Key Laboratory of Engineering Plastics and Beijing National Laboratory for Molecular Science, Institute of Chemistry, Chinese Academy of Sciences, Beijing 100190, China; weiyou@iccas.ac.cn (W.Y.); myanping@iccas.ac.cn (Y.M.)

**Keywords:** zinc complexes, ring-opening polymerization, polylactides

## Abstract

The model precatalyst *sp^3^*- and *sp^2^*-N dinitrogen-coordinated zinc–heteroimidazole has been used as an efficient catalyst for the ring-opening polymerization of cyclic esters. Subsequent to our exceptional active 5,6,7-trihydroquinolin-8-amine-zinc catalysts for the ring-opening polymerization (ROP) of *ε*-caprolactone, various pyridine-fused cycloalkanones (ring size from five to eight) are developed for the correspondent fused amine–pyridine derivatives and their zinc–heteroimidazole chloride complexes **Zn1**–**Zn8** (**L**ZnCl_2_) bearing *N*-diphenylphosphinoethyl pendants. Activated with two equivalents of LiN(SiMe_3_)_2_, the title zinc complexes efficiently promote the ROP of *L*-lactide (*L*-LA) in situ; among them, **Zn4**/2Li(NSiMe_3_)_2_ catalyzed 500 equivalent *L*-LA at 80 °C with 92% conversion in 5 min (TOF: 5520 h^−1^). Under the same conditions, the catalytic efficiency for the ROP of *rac*-LA by **Zn1**–**Zn8**/2Li(NSiMe_3_)_2_ was slightly lower than that for *L*-LA (highest TOF: 4440 h^−1^). In both cases, cyclooctyl-fused pyridyl–zinc complexes exhibited higher activity than others, while the cycloheptyl-fused zinc complexes showed the lowest activity. The microstructure analysis of the polymers showed they possessed a linear structure capped with CH_3_O as major and cyclic structure as minor. In this work, all the ligands and zinc complexes were well characterized by ^1^H/^13^C/^31^P NMR, FT-IR spectroscopy as well as elemental analysis.

## 1. Introduction

Aliphatic polyesters, such as polylactide (PLA) and polycaprolactone (PCL), being environment-friendly materials with good biocompatibility and biodegradability, have attracted considerable attention in the past decades [1,2]. The polycondensation of lactic acid and the ROP of lactides are two major processes in the formation of PLAs [3]. The molecular weights of the PLAs obtained through condensation are generally low, so ROP is extensively investigated for PLA production in order to take advantage of their useful properties [4]. Metal-complex precatalysts lead to the ROP of lactides(LA) with not only high efficiency but also the formation of well-defined PLAs [5,6,7]. Currently, tin(II) 2-ethylhexanoate is the most popular precatalyst for the industrial production of PLAs via ROP [8]; however, due to the potential cytotoxicity of tin, there are concerns about its use for PLA production, which is a limitation [9,10]. Therefore, the development of new metal complex catalysts for the ROP of LA based on non-toxic and environmental-friendly metals is in high demand [11,12,13,14]. As an essential element in the human body, as well as having low toxicity for animals and a low cost [15,16], zinc complexes have become a promising candidate for the ROP of cyclic esters. Targeting efficient zinc catalysts for the ROP of LA, various ligands have been explored such as β-diketiminate [17,18,19,20], phenolate [21,22,23,24,25,26], Schiff bases [27,28,29,30,31], bis(pyrazolyl)amide [32,33], *N*-heterocyclic carbenes [34,35,36] and guanidines [37,38,39]. The stereochemical attributes of the ligands are useful factors in controlling the micro-structure and properties of the resultant PLAs [40].

**Scheme 1:**
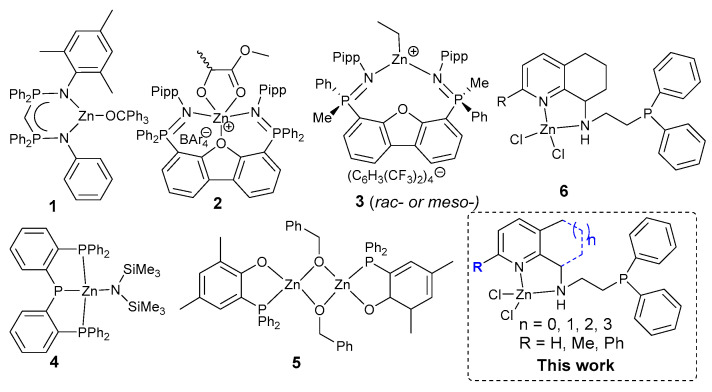
Zinc complexes containing phosphorus atoms for the ROP of lactides.

As described above, the ligands of zinc-complex precatalysts commonly contain nitrogen and/or oxygen donors [17,18,19,20,21,22,23,24,25,26,27,28,29,30,31,32,33,34,35,36,37,38,39,40]. In addition, there have been a few reports on the ROP of LA with zinc complexes containing a phosphorus group. For example, zinc complex **1** (Scheme 1), including a bis(phosphinimino)methyl ligand, catalyzed 100 equivalent rac-LA with a > 95% conversion rate within 2 h in toluene at 60 °C [41]. A series of cationic zinc complexes (**2** and **3**, Scheme 1) supported by bis(phosphinimine) ligands performed well in the ROP of *rac*-LA [42,43,44,45]. Zinc complex **2** (Scheme 1) achieved the ROP of *rac*-LA with 90% conversion in 50 min at ambient temperature at the molar ratio of [*rac*-LA]/[**2**] = 200:1, characterized by a slightly hetero-enriched microstructure [polymer tacticity (P_r_) = 0.63] [42]. Meanwhile, the cationic alkyl zinc complexes *rac*-**3** and *meso*-**3** (Scheme 1), as isolatable stereoisomers, individually catalyzed 1000 equivalent rac-LA monomers at 40 °C, resulting in PLAs with 89.6% conversion by *rac*-**3** in 4 h and 95.3% conversion by *meso*-**3** in 3.8 h, more importantly observing a higher molecular weight in PLA of 3.90 × 10^5^ g/mol [45]. Moreover, zinc complex **4** (Scheme 1), bearing a diarylphosphido-diphosphine, also efficiently initiated the ROP of *L*-LA with a 91% yield in the presence of isopropyl alcohol in toluene within 20 min with [*L*-LA]/[**4**] = 200 [46]. The extensive copolymerization of *L*-LA and *ε*-caprolactone (*ε*-CL) proceeded smoothly at 110 °C for random copolymers [47]. Another P^O-phosphinophenolate zinc complex **5** (Scheme 1) polymerized 100 equivalents of *rac*-LA with a 97% conversion rate in CH_2_Cl_2_ after 2 h and showed a good capability of catalyzing the homo(co)-polymerization of *L*-LA or *ε*-CL and trimethylene carbonate (TMC), achieving PCL/PTMC-*co*-PLLA co-polymers and PTMC-*co*-PCL-*co*-PLLA terpolymers [48].

Using the soft donor abilities of phosphorus-based ligands [49,50], a series of zinc complexes containing N^N^P ligands (**6**, Scheme 1) were evaluated for the ROP of ε-CL [51], achieving 90% conversion at high molar ratio of [ε-CL]/[Zn] = 5000:1 within 2 min, indicating a remarkable TOF of 1.35 × 10^5^ h^−1^; meanwhile, their iron analogues also performed well for the ROP of ε-CL (TOF up to 8.82 × 10^3^ h^−1^), producing high-molecular-weight PCL (Mn up to 2.43 × 10^5^ g/mol), demonstrating an exceptional model of iron-complex precatalysts [52]. Subsequently, a series of zinc complexes bearing cycloalkyl-fused pyridines with ring sizes tuned from five to eight within their backbones are prepared and achieve good activity for the ROP of lactides (*L*-LA and *rac*-LA) under different parameters (time, temperature, solvent or molar ratio of monomer), indicating the significant influence of the ring size of fused cycloalkyls and of the R group on the ortho-position of pyridine on catalytic performance.

## 2. Results and Discussion

### 2.1. Synthesis and Characterization of **L1**–**L8** and Their Zn (II) Complexes **Zn1**–**Zn8**

**Scheme 2:**
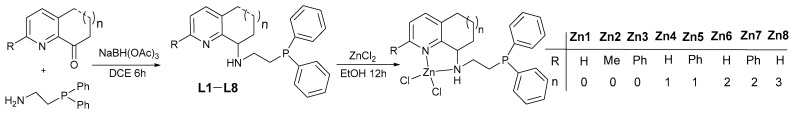
Synthesis of **L1**–**L8** and Zn (II) complexes **Zn1**–**Zn8**.

A series of ligands **L1**–**L8** of monocycloalkyl (ring size from five to eight)-fused pyridine-bearing pendant N-diphenylphoshinoethyl groups, N-(Ph_2_PCH_2_CH_2_)-2-RC_7_H_7_N-8-NH (R = H **L1**, R = Ph **L2**, R = Me **L3**), N-(Ph_2_PCH_2_CH_2_)-2-RC_9_H_9_N-8-NH (R = H **L4**, R = Ph **L5**), N-(Ph_2_PCH_2_CH_2_)-2-RC_10_H_11_N-8-NH (R = H **L6**, R = Ph **L7**), N-(Ph_2_PCH_2_CH_2_)-2-RC_11_H_13_N-8-NH (R = H **L8**), were prepared according to the literature (as shown in Scheme 2) [53,54,55]. The Zn(II) complexes **Zn1**–**Zn8** were obtained by treating the ligands **L1**–**L8** with zinc(II) chloride in ethanol at room temperature for 12 h, respectively. All novel compounds were confirmed by ^1^H, ^13^C and ^31^P NMR (shown in Figures S1–S36), FT-IR spectroscopy and elemental analysis. The ^31^P NMR spectrum of ligands showed one signal around −20 ppm, while their corresponding zinc complexes showed a shift around −22 ppm. These little shifts changes in the ^31^P NMR spectra indicated little environmental change around phosphine, and also suggesting the possible dissociation of phosphine to zinc. Subsequently, the structures of **Zn2**, **Zn3**, **Zn7** and **Zn8** were further determined by single-crystal X-ray diffraction, which agreed well with their ^31^P NMR spectra.

**Figure 1:**
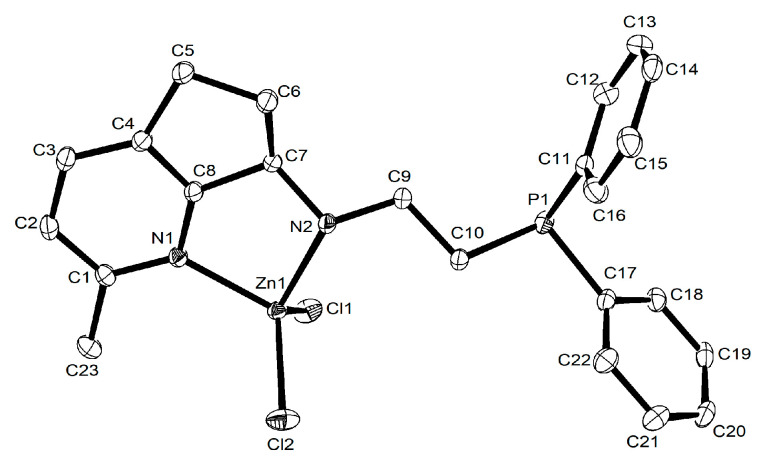
An ORTEP drawing of **Zn2** with thermal ellipsoids of 30% probability. All hydrogen atoms are omitted for clarity.

**Figure 2:**
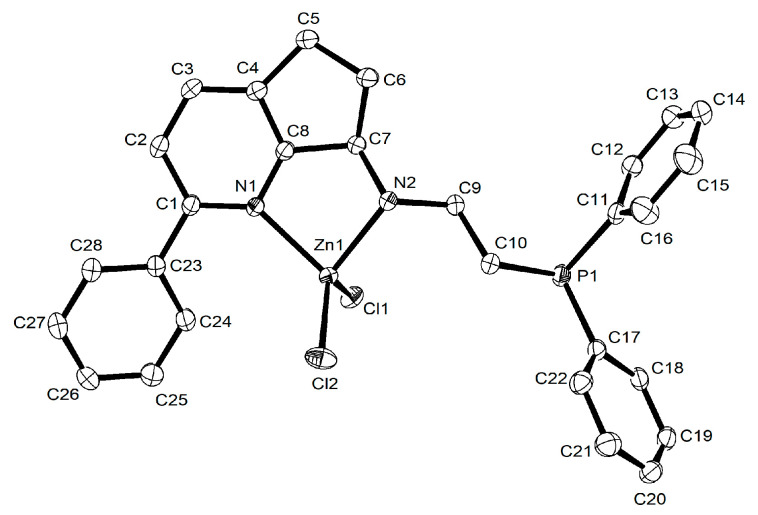
An ORTEP drawing of **Zn3** with thermal ellipsoids of 30% probability. All hydrogen atoms are omitted for clarity.

**Figure 3:**
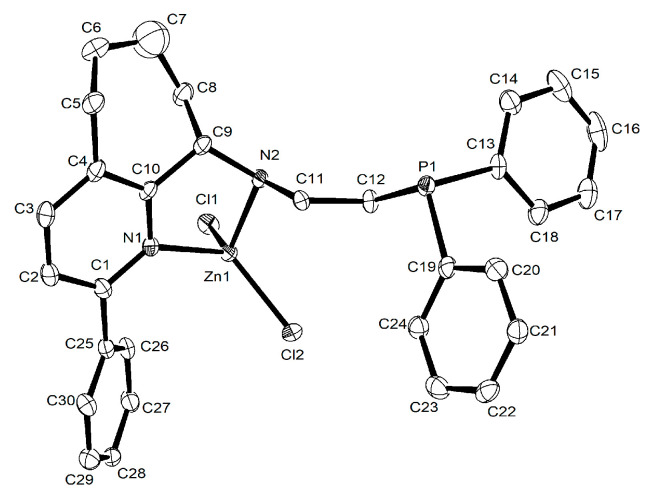
An ORTEP drawing of **Zn7** with thermal ellipsoids of 30% probability. All hydrogen atoms are omitted for clarity.

**Figure 4:**
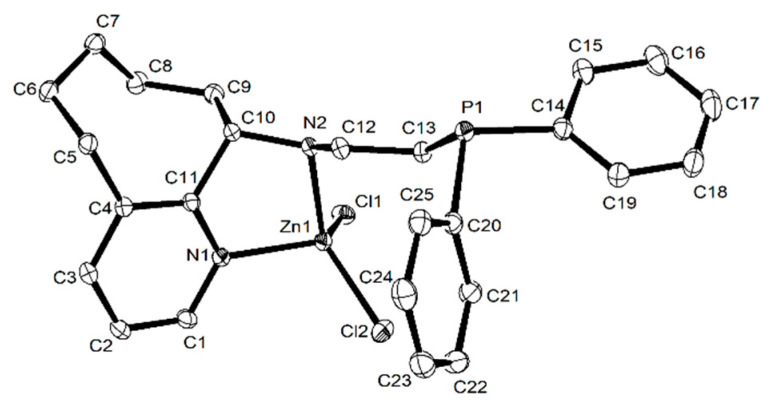
An ORTEP drawing of **Zn8** with thermal ellipsoids of 30% probability. All hydrogen atoms are omitted for clarity.

The single crystals of **Zn2**, **Zn3**, **Zn7** and **Zn8** suitable for X-ray determination were obtained by diffusing diethyl ether into a dichloromethane solution. Their crystal structures are depicted in Figure 1, Figure 2, Figure 3 and Figure 4, respectively, and the selected bond lengths and angles are summarized in Table 1. The molecular structures of **Zn2**, **Zn3**, **Zn7** and **Zn8** are similar, in which the zinc is four-coordinated by two nitrogen atoms and two chlorine atoms to form a distorted tetrahedral geometry. Similar to our previous reports [51], the phosphorus donor did not coordinate to zinc, which agreed well with the little shift in its ^31^P NMR spectra.

The bond distances between the Zn (II) and Cl atoms vary between 2.2018(5) and 2.230(2) Å and the Zn-N bond lengths vary between 2.0442(13) and 2.1162(16) Å. Notably, the bond lengths of the Zn-Cl bond are longer than those of the Zn-N bond, which is in accordance with previous reports [56,57]. As shown in Table 1, the bond length of the Zn-NH bond is longer than that of the Zn-N_quinoline_ bond in both the **Zn2** and **Zn8** structures [2.1091(15) vs. 2.0777(15); 2.0920(12) vs. 2.0442(13) Å], indicating more effective coordination by N_quinoline_ than NH; On the contrary, the bond length of Zn-NH in **Zn3** and **Zn7** is shorter than that of Zn–N_quinoline_. The different bond lengths between Zn-NH and Zn-N_quinoline_ indicate that the size of substituents and rings influence the molecular structure of the zinc complexes.

The R substituent and fused ring size also greatly affect the structure. For **Zn3**, **Zn5** and **Zn7**, all bearing the same R substituent of phenyl, the dihedral angle between the phenyl ring and pyridine plane is 26.49° in **Zn3** (five-ring), which is much smaller than that (43.07°) in **Zn7** (seven-ring) and that (48.74°) in **Zn5** (six-ring) [51], suggesting the great influence of cycloalkyl ring size. Another interesting observation is that, due to the torsion of different cycloalkyls, the distance from N(H) and P to the pyridine plane varied, as shown by 0.440 Å and 0.537 Å in **Zn3**, 0.703 Å and 1.365 Å in **Zn5** and 0.471 Å and −3.918 Å in **Zn7**, respectively, suggesting the increased distortion of the **Zn7** structure. In addition, regarding the distortion of the rings, the carbon C6 atoms of the cycloalkyl deviate from the pyridyl-based plane with a distance of 0.408 Å for **Zn2**, 0.348 Å for **Zn3**, 1.340 Å for **Zn7** and 1.400 Å for **Zn8**. These different distortions will have an effect on their catalytic activity.

### 2.2. Ring-Opening Polymerization (**ROP**) of L-LA by **Zn1-Zn8**/2LiN(SiMe_3_)_2_

In the beginning, **Zn6** was tested for the ROP of *L*-LA without any additional initiator, but there was no activity observed, considered the result of the stability of the Zn-Cl bond. According to our previous work [51], a binary system consisting of zinc complexes and two equivalents of LiN(SiMe_3_)_2_ exhibited extremely high activity toward the ring-opening polymerization of ɛ-CL in situ; here, a similar system was explored for the ROP of *L*-LA. Firstly, the effect of LiN(SiMe_3_)_2_ on the ROP of *L*-LA was investigated by using **Zn6** in toluene at a molar ratio of [LA]:[Zn] = 250:1 and within 10 min at 30 °C. The results showed that no polymer was obtained when using one equivalent LiN(SiMe_3_)_2_. However, when increasing the LiN(SiMe_3_)_2_ from one to two equivalents, the monomer conversion could reach 36% under the same conditions. In addition, the blank experiments showed 10% conversion of monomers was achieved using only two equivalents of LiN(SiMe_3_)_2_ as catalysts. Therefore, a catalyst system of **Zn6**/2LiN(SiMe_3_)_2_ was employed for the ROP of *L*-LA, and the polymerization results are collected in Table 2.

In the first instance, the effect of benzyl alcohol (BnOH) on polymerization was investigated with the molar ratio of [*L*-LA]:[Zn] = 250:1 at a temperature kept at 30 °C (runs 1–3, Table 2). The results indicated that the monomer conversion decreased from 36% to 11% when one equivalent benzyl alcohol was added. Then, the amount of benzyl alcohol was further increased to three equivalents, and the monomer conversion continued to decrease to only 8%. These results revealed that benzyl alcohol could not improve the monomer conversion in this catalytic system. In order to enhance the monomer conversion, the polymerization was screened from 10 min to 60 min under the same conditions (runs 1 and 4–6, Table 2). The results showed that monomer conversion greatly increased from 36% in 10 min to 86% in 60 min with the extension of time, but the molecular weight decreased from 3.99 × 10^4^ to 1.65 × 10^4^ g/mol and the polydispersity became broader, suggesting the increased side reaction of transesterification during longer times. The comparison of the *M*_n_ values with the calculated values [*M*_n(calcd)_] shows large differences, which indicates that the polymerizations are not well controlled [59]. The kinetics of polymerization by **Zn6**, shown as a semilogarithmic plot, slightly deviates from the first order with the polymerization rate constant *k*_app_ as 0.03177 min^−1^ (LA/Zn = 250:1) or 0.02294 min^−1^ (LA/Zn = 250:0.5) (Figure S37 in Supplementary Materials).

Secondly, the molar ratio of [L-LA]:[Zn] was fixed at 250:1 and the run time at 10 min, and the influence of temperature on polymerization was also investigated (runs 1 and 7–10, Table 2). As the reaction temperature increased from 30 °C to 80 °C, the monomer conversion rate also increased gradually from 36% to 100%, indicating that higher temperatures favored the increase in the polymerization rate. At the same time, the polymer obtained from 50 °C and 80 °C possessed similar *M*_n_ values (M_n_ = 2.66–2.83 × 10^4^ g/mol) and molecular weight distributions (M_w_/M_n_ = 1.87–2.08); within this temperature range, the M_n_ values were all lower than the *M*_n(calcd)_ values. This may be explained by the efficiency of active species and the effect of transesterification during polymerization [60]. Moreover, the molecular weight distribution of all obtained polymers was broad, suggesting more side reactions of transesterification at higher temperatures [61,62,63].

Subsequently, in order to explore the role of various solvents for the ROP of *L*-LA, the polymerizations were carried out in dichloromethane at 30 °C with THF and 60 °C with n-hexane (runs 11–13, Table 2). When THF was used, high monomer conversion of up to 92% was achieved at [*L*-LA]:[Zn] = 250:1 within 10 min. However, when dichloromethane and n-hexane were used, the monomer conversion was extremely low, at 0 and 8%, respectively. The reason for this may be related to the coordination competition between the monomer and solvent for the active zinc centers [64,65].

Fixing the temperature at 80 °C and run time at 10 min, increasing the molar ratio of [*L*-LA]:[Zn] from 250:1 to 1000:1 led to a slightly decrease in monomer conversion from 100% to 89% (runs 10, 14, 15, Table 2), but the molecular weights of the polymer were very similar (from 2.46 to 2.66 × 10^4^ g/mol), which were much lower than *M*_n(calcd)_. Furthermore, the effect of the amount of toluene was also explored at a high molar ratio of [*L*-LA]:[Zn] = 1000:1 in 10 min at 80 °C (runs 15–17, Table 2). When increasing the toluene volume from 1 mL to 5 mL, the monomer conversion gradually decreased from 89% to 65% and the molecular weight of the polymer slightly decreased from 2.61 × 10^4^ g/mol to 2.02 × 10^4^ g/mol. This may be attributed to the deactivation of some active species caused by more impurities in toluene [66]. Moreover, the reaction time was reduced from 10 min to 5 min at 80 °C with the molar ratio of [*L*-LA]:[Zn] = 500:1, and the monomer conversion also decreased from 96% to 63% (runs 15 vs. 18, Table 2). The average molecular weight of the obtained polymer decreased from 2.61 × 10^4^ g/mol to 1.86 × 10^4^ g/mol.

**Figure 5:**
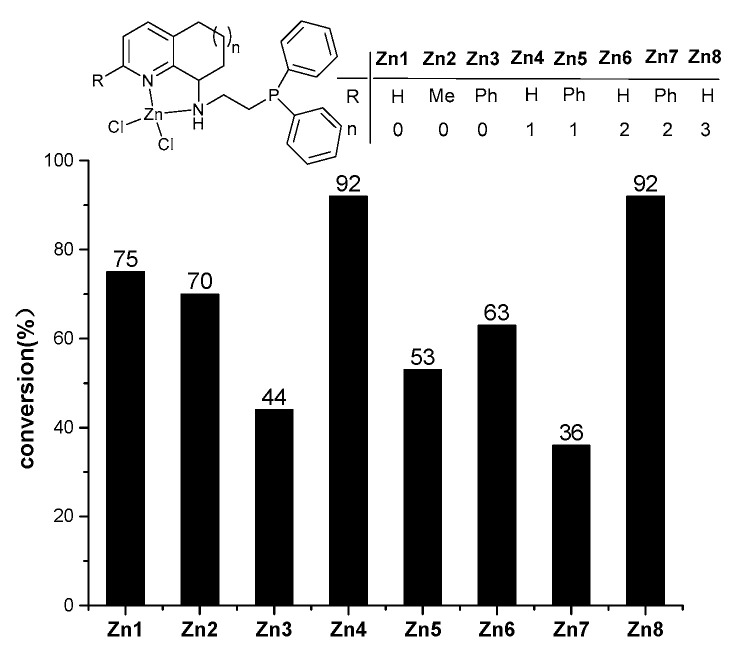
Comparison of the monomer conversion (*L*-LA) achieved using **Zn1**–**Zn8**/2LiN(SiMe_3_)_2_ (runs 18, 19–25, Table 2).

With the [*L*-LA]:[Zn] ratio kept at 500:1 and temperature and time at 80 °C and 5 min, respectively, all zinc complexes **Zn1**–**Zn8** were evaluated for the ROP of *L*-LA by using pre-treatment with two equivalents of LiN(SiMe_3_)_2_, and the results are shown in runs 18, 19–25 (Table 2). The data revealed that the catalytic system **Zn1**–**Zn8**/2LiN(SiMe_3_)_2_ can efficiently catalyze the ROP of *L*-LA, in which the catalytic efficiency is related with the size of the substituent group (R) and the size of the pyridyl-fused ring (shown in Figure 5).

According to Figure 5, complexes **Zn4** (six-ring) and **Zn8** (eight-ring) show higher monomer conversion rates than the other complexes, both exceeding 90% (TOF = 5.52 × 10^3^ h^−1^), much higher than those of the other zinc complexes. The R also greatly affected the activity, as shown by the following activity order: **Zn1** (75%, H) > **Zn2** (70%, Me) > **Zn3** (44%, Ph); **Zn4** (92%, H) > **Zn5** (53%, Ph); **Zn6** (63%, H) > **Zn7** (36%, Ph). This indicated that the smaller substituents favored the polymerization. The fused ring size also greatly affected the activity, as shown by the following activity order: R = H, **Zn4** (six-ring) = **Zn8** (eight-ring) > **Zn1** (75%, five-ring) > **Zn6** (63%, seven-ring); R = Ph, **Zn5** (53%, six-ring) > **Zn3** (44%, five-ring) > **Zn7** (36%, seven-ring). Thus, **Zn7** and **Zn6** displayed the lowest activity, and the reason is probably due to the more distorted fused seven-ring structures in **Zn7** and **Zn6**. The polymer obtained by using **Zn4** possessed the highest molecular weight (M_n_ = 3.9 × 10^4^ g/mol).

**Figure 6:**
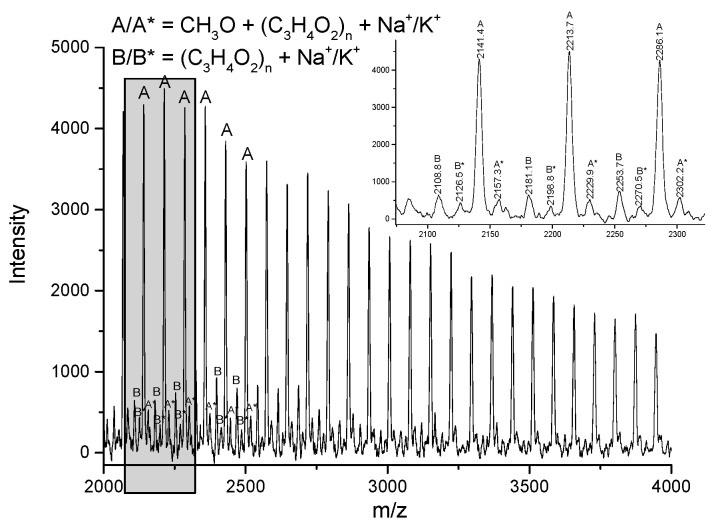
MALDI–TOF mass spectrum of the PLLA obtained using **Zn6**/2LiN(SiMe_3_)_2_ (run 18, Table 2).

**Figure 7:**
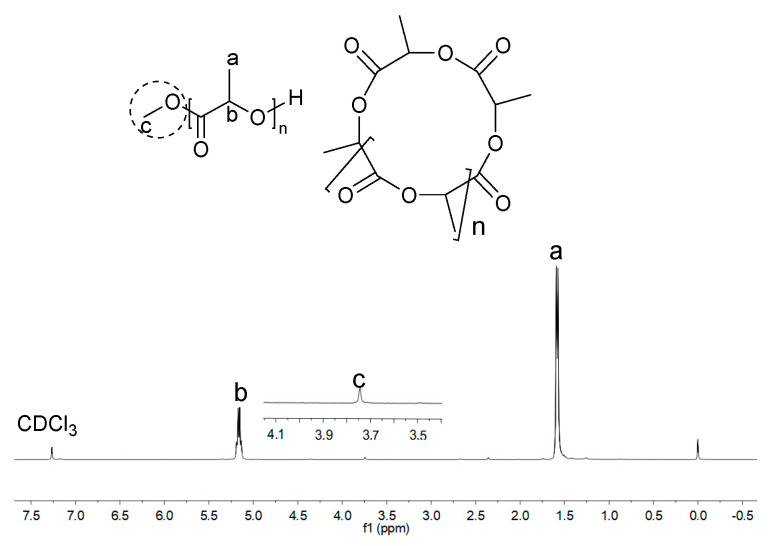
^1^H NMR spectrum of the PLLA obtained using **Zn6**/2LiN(SiMe_3_)_2_ (run 18, Table 2).

In order to gather more information about the mechanism of the ROP of L-LA, the obtained PLLA (run 18, Table 2) was characterized by a ^1^H NMR and MALDI-TOF spectrum. The MALDI-TOF mass spectrum revealed two families of peaks (as shown in Figure 6): both A/A* and B/B* were separated by an *m*/*z* of 72, in which the major peaks A/A* can be ascribed to CH_3_OH + (C_3_H_4_O_2_)_n_ + Na^+^/K^+^ and the minor peaks B/B* are assignable to (C_3_H_4_O_2_)_n_ + Na+/K+. The ^1^H NMR spectrum clearly showed the presence of CH_3_O protons around δ 3.75 ppm (shown in Figure 7), which may come from the methanolysis of the polymer chain during the quenching process [60,67,68]. In addition, there was no signal of -N(SiMe_3_)_2_ in either the ^1^H NMR or MALDI-TOF spectra, which was consistent with a previous report in the literature [51].

**Scheme 3:**
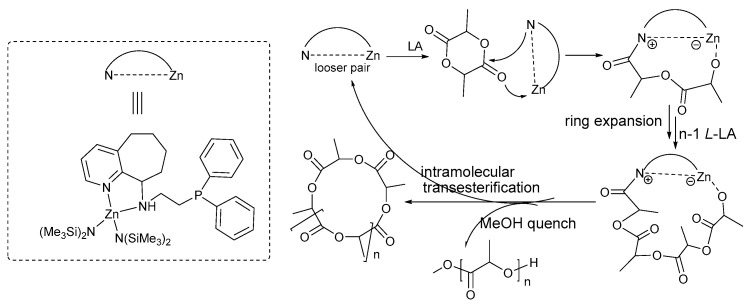
Possible mechanistic pathways for the ROP of *L*-LA using **Zn6**/2LiN(SiMe_3_)_2_.

Based on the above results, the mechanism of the ROP of *L*-LA catalyzed by **Zn1**–**Zn8**/2LiN(SiMe_3_)_2_ was proposed, as shown in Scheme 3. It is assumed that a Lewis pair will form during the ROP process and can be ‘loosened up’ at higher temperatures to form a zwitterionic intermediate. Subsequently, this ‘loose’ pair can initiate the polymerization, causing the ring to expand to form the cyclic macrolactone metal ring; this mechanism has also been reported in the literature [51,69,70]. Finally, methanol was added to terminate the polymerization reaction, and a linear polymer capped with a methoxyl group was obtained [71]. Furthermore, its minor cyclic structure may be due to intramolecular transesterification.

### 2.3. Ring-Opening Polymerization of rac-LA by **Zn1–Zn8**/2LiN(SiMe_3_)_2_

Under the conditions of the molar ratio of the monomer to the catalyst of 500:1, polymerization temperature of 80 °C and reaction time of 5 min, the catalytic performance of **Zn1**–**Zn8**/2LiN(SiMe_3_)_2_ for the ROP of *rac*-LA was also investigated. The polymerization results are shown in Table 3.

**Figure 8:**
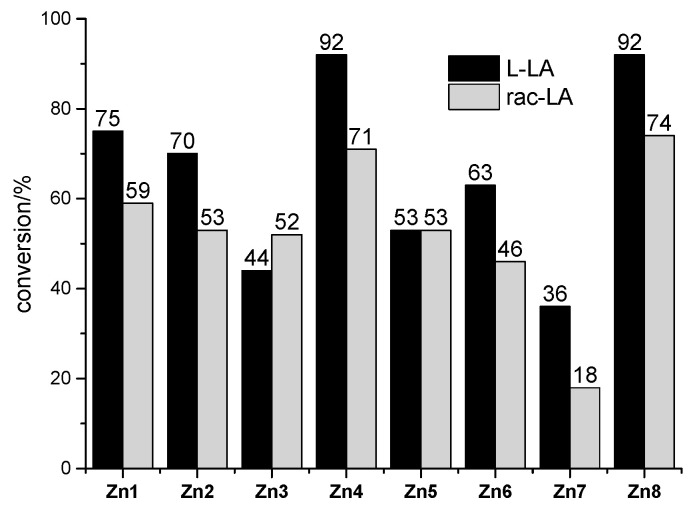
Comparison of the monomer conversions (*L*-LA and *rac*-LA) achieved using **Zn1**–**Zn8**/2LiN(SiMe_3_)_2_.

The results showed that **Zn1**–**Zn8**/2LiN(SiMe_3_)_2_ also displayed good efficiency for the ROP of *rac*-LA but without stereoselectivity and all the resultant polymers were amorphous. Both the substituent and ring size of the ligand greatly influenced the activity, and the order of activity is as follows: **Zn1** (59%, R = H) > **Zn2** (53%, R = Me) > **Zn3** (52%, R = Ph); **Zn4** (71%, R = H) > **Zn5** (53%, R = Ph); **Zn6** (46%, R = H) > **Zn5** (18%, R = Ph). **Zn8**, bearing a small substituent group with an eight-membered ring, exhibited the highest activity (TOF = 4.44 × 10^3^ h^−1^), higher than **Zn1**–**Zn7** (TOF range: 1.08–4.26 × 10^3^ h^−1^). Additionally, the catalytic efficiency of the Zn/2LiN(SiMe_3_)_2_ catalytic system for the ROP of *rac*-LA was generally lower than that for the ROP of *L*-LA (Figure 8).

The microstructure of poly(*rac*-LA) (run 1, Table 3) was also studied by MALDI-TOF and ^1^H NMR spectra. The MALDI-TOF mass spectrometry showed two families of A/A* and B/B* (as shown in Figure S38), in which the major family peaks A/A* were attributed to CH_3_OH + (C_3_H_4_O_2_)_n_ + Na^+^/K^+^, as the visible linear structure with a methoxy end group, while the minor family peaks B/B* were assigned as the cyclic structure (C_3_H_4_O_2_)_n_ + Na^+^/K^+^. According to the ^1^H NMR spectrum (as shown in Figure S39), the signal at around 3.75 ppm of the CH_3_O protons appeared to further confirm the MALDI-TOF analysis. Therefore, the mechanism is similar to that for the ROP of *L*-LA. All the obtained polymers were sticky. In order to exemplify their stereoselectivity, they were further characterized by using decoupling ^1^H NMR (shown in Figure S40) and results showed that their *P*m values varied approximately between 0.30 and 0.35.

## 3. Materials and Methods

### 3.1. General Considerations

All operations of moisture- and air-sensitive chemicals were carried out under an atmosphere of high-purity nitrogen using standard Schlenk techniques and a N_2_-filled glovebox. LiN(SiMe_3_)_2_ (1.0 M in THF) was purchased from Aldrich (San Diego, CA, USA) and used as received. Toluene, n-hexane, tetrahydrofuran and diethyl ether were dried by refluxing over sodium benzophenone, distilled under N_2_ and stored over activated molecular sieves (4 Å) for 24 h in a glovebox prior to use. Dichloromethane was dried over CaH_2_ for 48 h, distilled under N_2_ and stored over activated molecular sieves (4 Å) in a glovebox prior to use. L-lactide and rac-lactide were purchased from TCI and used as received without further purification. And other reagents were obtained from common commercial suppliers.

All NMR spectra were recorded with Bruker Avance III 400 or Bruker Avance III 400 HD spectrometers (400 MHz for ^1^H NMR, 100 MHz for ^13^C NMR) at room temperature. The chemical shifts were related to the position of the NMR signal using tetramethylsilane (TMS) as a reference. And ^31^P NMR spectra were measured on a Bruker Avance II + 400 instrument. Elemental analyses were performed with a Flash EA 1112 microanalyzer, while IR spectra were conducted using a Perkin-Elmer System 2000 FT-IR spectrometer (Thermo Flash Smart, Beijing, China). MALDI-TOF MS analysis was performed with a Bruker Ultraflex mass spectrometer. For MALDI MS analysis, mass spectra were acquired with a SmartBeam laser (355 nm, Bruker, Bremen, Germany) operating at 200 Hz with a laser focus of 50 μm. The device parameters for MALDI MS were chosen as follows: plate offset voltage, 19 kV; deflector detector voltage, 20 kV. Data were processed using DateAnalysis 3.0 (Bruker Daltonics,). GPC analysis was performed using a system composed of a 390-LC multidetector (MDS), a 209-LC pump injection module (PIM) and a PL-GPC 50 plus instrument. THF was used as the eluent (flow rate: 1 mL/min, at 40 °C) and the molecular weights and molecular weight distributions were calculated using polystyrene as the standard.

### 3.2. Synthesis and Characterization of Ligands and Zinc Complexes

#### 3.2.1. 2-(Diphenylphosphino)ethanamine, Ligands (**L1**–**L8**) and Zinc Complexes (**Zn4**, **Zn5**) Were Synthesized as Previously Reported [51,53,54,55]

**2-(diphenylphosphino)ethanamine**. In a 250 mL double-mouth round-bottom flask filled with nitrogen, ^t^BuOK (12.03 g, 105 mmol) and diphenylphosphine (9.63 g, 52.6 mmol) were dissolved in 100 mL dry THF and the solution was stirred for 1 h at ambient temperature. Then, 2-chloroethylamine hydrochloride (6.58 g, 55.6 mmol) was added to the mixture and heated to reflux for 20 h at 80 °C. During the reaction. After cooling to room temperature, THF was removed under reduced pressure. First, 10% HCl was added to make the solution strongly acidic, and then the solution was washed three times with toluene. Secondly, the mixture was extracted three times with toluene after beingneutralized with 10% NaOH. Finally, the resulting toluene solution was washed three times with saturated NaCl and dried with MgSO_4_ to give a yellow oily liquid (9.4 g, 77% yield). ^1^H NMR (400 MHz, CDCl_3_, TMS): δ 7.47–7.38 (m, 4H), 7.35–7.28 (m, 6H), 2.88–2.80 (m, 2H), 2.24 (t, *J* = 8.0 Hz), 1.57 (s, 2H). ^13^C NMR (100 MHz, CDCl_3_, TMS): δ 137.16, 13.64, 132.81, 132.62, 130.78, 130.66, 128.96, 128.84, 128.77, 128.58, 128.51, 38.81, 38.59, 30.91, 30.78. ^31^P NMR (162 MHz, CDCl_3_, TMS): δ 22.03.

**N-(2-(diphenylphosphaneyl)ethyl)-6,7-dihydro-5H-cyclopenta[b]pyridin-7-amine (L1).** 5,6-dihydro-7H-cyclopenta[b]pyridin-7-one (0.5 g, 3.85 mmol), 2-(diphenylphosphino)ethanamine (1.15 g, 5 mmol) and sodium triacetoxyborohydride (1.7 g, 8.05 mmol) were dissolved in 50 mL 1,2-dichloroethane (DCE). The rection mixture was stirred for 6 h at ambient temperature under a nitrogen atmosphere. After the reaction, the mixture was quenched with saturated NaHCO_3_ and the organic layer was extracted three times with ethyl acetate. Then, the mixture was dried with MgSO_4_ and the solvent was removed with a rotary evaporator. The crude product was further purified by chromatography on silica gel with petroleum ether/ethyl acetate (5:1, *v*/*v*) to yield the product as a green oily liquid (0.7 g, 52% yield). ^1^H NMR (400 MHz, CDCl_3_, TMS): δ 8.37 (d, *J* = 4.9 Hz, 1H), 7.50 (d, *J* = 7.5 Hz, 1H), 7.48–7.39 (m, 4H), 7.38–7.28 (m, 6H), 7.09–7.06 (m. 1H), 4.18 (t, *J* = 7.1 Hz), 3.02–2.74 (m, 4H), 2.43–2.33 (m, 3H), 1.91 –1.79 (m, 1H). ^13^C NMR (100 MHz, CDCl_3_, TMS): δ 163.41, 145.81, 137. 49, 137.41, 137.37, 137.29, 135.54, 131.88, 131.70, 131.69, 131.65, 131.52, 127.63, 127.51, 127.47, 127.44, 127.40, 127.38, 121.19, 61.73, 43.97, 30.48, 28.19, 28.07, 27.12. ^31^P NMR (162 MHz, CDCl_3_, TMS): δ 20.74. FT-IR (cm^−1^): 3049 (m), 2934 (m), 1671 (w), 1583 (m), 1480 (m), 1432 (m), 1423 (m), 1330 (m), 1264 (m), 1182 (w), 1092 (m), 1023 (m), 867 (w), 790 (m), 736 (s), 693.18 (s). Anal. Calcd for C_22_H_23_N_2_P (1/2H_2_O): C, 74.35; H, 6.81; N, 7.88. Found: C, 73.91; H, 6.86; N, 7.57.

**N-(2-(diphenylphosphaneyl)ethyl)-2-methyl-6,7-dihydro-5H-cyclopenta[b]pyridine-7-amine (L2).** The synthesis of **L2** followed the same procedure as that of **L1**, except that 2-methyl-5,6-dihydro-7H-cyclopenta[b]pyridin-7-one (1.13 g, 7,7 mmol) was used to yield the product as a green oily liquid (0.94 g, 34% yield). ^1^H NMR (400 MHz, CDCl_3_, TMS): δ 7.45–7.37 (m. 5H), 7.33–7.26 (m, 6H), 6.92 (d, *J* = 7.7 Hz, 1H), 4.13 (t, *J* = 6.8 Hz, 1H), 2.98–2.69 (m, 4H), 2.50 (s, 3H), 2.38–2.29 (m, 3H), 2.24 (s, 1H), 1.91–1.76 (m, 1H). ^13^C NMR (100 MHz, CDCl_3_, TMS): δ 162.88, 155.54, 137.59, 137.46, 137.33, 132.27, 131.91, 131.85, 131.73, 131.65, 131.47, 127.63, 127.46, 127.42, 127.39, 127.36, 120.74, 61.87, 43.98, 43.76, 30.44, 28.27, 28.15, 26.80, 23.05. ^31^P NMR (162 MHz, CDCl_3_, TMS): δ 18.57. FT-IR (cm^−1^): 3049 (m), 2929 (m), 1669 (w), 1584 (m), 1480 (w), 1431 (s), 1327 (m), 1260 (m), 1223 (m), 1181 (m), 1151 (m), 1094 (m), 1023 (m), 833 (w), 798 (m), 737 (s), 691 (s). Anal. Calcd for C_23_H_25_N_2_P (2/3 H_2_O): C, 74.17; H, 7.13; N, 7.52. Found: C, 74.36; H, 7.06; N, 7.30.

**N-(2-(diphenylphosphaneyl)ethyl)-2-phenyl-6,7-dihydro-5H-cyclopenta[b]pyridin-7-amine (L3).** The synthesis of **L3** followed the same procedure as that for **L1**, except that 2-phenyl-5,6-dihydro-7H-cyclopenta[b]pyridin-7-one (2.10 g, 10 mmol) yielded the product as a green oily liquid (1.34 g, 32% yield). ^1^H NMR (400 MHz, CDCl_3_, TMS): δ 7.95 (d, *J* = 7.4 Hz, 2H), 7.57–7.43 (m, 9H), 7.34–7.29 (m, 6H), 4.23 (t, *J* = 7.2 Hz, 1H), 3.09–2.78 (m, 4H), 2.47–2.38 (m, 3H), 2.20 (s, 1H), 1.95–1.85 (m, 1H). ^13^C NMR (100 MHz, CDCl_3_, TMS): δ 163.65, 155.08, 138.69, 137.56, 137.50, 137.44, 137.37, 133.95, 132.20, 131.89, 131.70, 131.52, 127.60, 127.50, 127.47, 127.45, 127.41, 127.38, 125.90, 118.29, 61.80, 44.09, 43.87, 30.77, 28.35, 28.23, 26.89. ^31^P NMR (162 MHz, CDCl_3_, TMS): δ 20.35. FT-IR (cm^−1^): 3053 (m), 2932 (m), 1584 (m), 1480 (w), 1431 (s), 1327 (m), 1260 (m), 1223 (m), 1181 (m), 1151 (m), 1094 (m), 1023 (m), 833 (w), 798 (m), 737 (s), 691 (s). Anal. Calcd for C_28_H_27_N_2_P (1/2 H_2_O): C, 77.94; H, 6.54; N, 6.49. Found: C, 77.78; H, 6.53; N, 6.50.

**N-(2-(diphenylphosphaneyl)ethyl)-5,6,7,8-tetrahydro-quinolin-8-amine (L4).** The synthesis of **L4** followed the same procedure as that for **L1**, except that 6,7-dihydroquinolin-8(5H)-one (1.13 g, 7.7 mmol) yielded the product as a yellow oily liquid (1.66 g, 60% yield). ^1^H NMR (400 MHz, CDCl_3_, TMS): δ 8.42 (d, *J* = 4.2 Hz, 1H), 7.53–7.45 (m, 4H), 7.41–7.29 (m, 7H), 7.11–7.04 (m, 1H), 3.82 (t, *J* = 5.8 Hz, 1H), 3.03–2.75 (m, 4H), 2.73 (s, 1H), 2.42 (t, *J* = 7.8 Hz, 2H), 2.15–1.94 (m, 2H), 1.81–1.66 (m, 2H). ^13^C NMR (100 MHz, CDCl_3_, TMS): δ 157.26, 146.81, 138.63, 138.53, 138.50, 138.41, 136.85, 132.92, 132.73, 132.66, 132.48, 132.37, 128.59, 128.43, 128.40, 128.37, 128.34, 121.79, 57.79, 44.52, 44.30, 29.24, 29.12, 28.80, 28.52, 19.57. ^31^P NMR (162 MHz, CDCl_3_, TMS): δ 20.38.

**N-(2-(diphenylphosphaneyl)ethyl)-2-phenyl-5,6,7,8-tetra-hydroquinolin-8-amine (L5).** The synthesis of **L5** followed the same procedure as that for **L1**, except that 2-phenyl-6,7-dihydroquinolin-8(5H)-one (1.71 g, 7.7 mmol) yielded the product as a yellow oily liquid (1.98 g, 60% yield). ^1^H NMR (400 MHz, CDCl_3_, TMS): δ 7.99–7.91 (m, 2H), 7.49–7.21 (m, 15H), 3.78 (t, *J* = 6.5 Hz, 1H), 2.99–2.64 (m, 5H), 2.42–2.33 (m, 2H), 2.14–1.60 (m, 2H), 1.73–1.60 (m, 2H). ^13^C NMR (100 MHz, CDCl_3_, TMS): δ 155.97, 153.27, 138.47, 137.73, 137.60, 137.49, 136.58, 131.84, 131.77, 131.65, 131.58, 129.72, 127.57, 127.51, 127.42, 127.35, 125.71, 117.47, 57.07, 43.54, 43.33, 28.51, 28.39, 28.07, 27.58, 19.02. ^31^P NMR (162 MHz, CDCl_3_, TMS): δ 20.44.

**N-(2-(diphenylphosphaneyl)ethyl)-6,7,8,9-tetrahydro-5H-cyclohepta[b]pyridin-9-amine (L6).** The synthesis of **L6** followed the same procedure as that for **L1**, except that 5,6,7,8-tetrahydro-9H-cyclohepta[b]pyridin-9-one (1.23 g, 7.7 mmol) yielded the product as a yellow oily liquid (1.51 g, 52% yield). ^1^H NMR (400 MHz, CDCl_3_, TMS): δ 8.29 (d, *J* = 4.6 Hz, 1H), 7.44–7.34 (m, 4H), 7.32–7.20 (m, 7H), 6.98 (t, *J* = 6.0 Hz, 1H), 3.84 (d, *J* = 9.2 Hz, 1H), 2.89–2.58 (m, 4H), 2.39–2.27 (m, 3H), 1.95–1.88 (m, 2H), 1.74–1.63 (m, 2H), 1.53–1.34 (m, 2H). ^13^C NMR (100 MHz, CDCl_3_, TMS): δ 161.12, 144.75, 135.79, 131.81, 131.59, 127.45, 127.39, 127.38, 127.32, 127.31, 120.46, 62.27, 44.14, 43.93, 33.20, 33.53, 28.34, 28.23, 27.62, 26.34. ^31^P NMR (162 MHz, CDCl_3_, TMS): δ 20.40. FT-IR (cm^−1^): 3049 (w), 2921 (m), 1572 (m), 1476 (w), 1430 (m), 1337 (w), 1260 (m), 1090 (m), 1019 (m), 792 (m), 737 (s), 693 (s). Anal. Calcd for C_24_H_27_N_2_P (2/3 H_2_O): C, 74.59; H, 7.39; N, 7.25. Found: C, 74.43; H, 7.48; N, 7.08.

**N-(2-(diphenylphosphaneyl)ethyl)-2-phenyl-6,7,8,9-tetra-hydro-5H-cyclohepta[b]pyridin-9-amine (L7).** The synthesis of **L7** followed the same procedure as that for **L1**, except that 2-phenyl-5,6,7,8-tetrahydro-9H-cyclohepta[b]pyridin-9-one (1.74 g, 7.7 mmol) yielded the product as a yellow solid (2.16 g, 62% yield). ^1^H NMR (400 MHz, CDCl_3_, TMS): δ 8.02 (d, *J* = 7.4 Hz, 2H), 7.53–7.32 (m, 9H), 7.28–7.19 (m, 6H), 3.91 (d, *J* = 9.3 Hz, 1H), 2.90–2.68 (m, 4H), 2.50–2.31 (m, 2H), 2.09–1.94 (m, 2H), 1.91–1.69 (m, 2H), 1.56–1.32 (m, 2H). ^13^C NMR (100 MHz, CDCl_3_, TMS): δ 161.54, 153.16, 139.48, 138.75, 138.68, 138.63, 138.55, 137.76, 135.35, 132.80, 132.62, 132.59, 128.60, 128.48, 128.45, 128.35, 126.71, 117.91, 63.07, 45.50, 45.28, 34.29, 33.90, 29.45, 29.35, 29.20, 27.41. ^31^P NMR (162 MHz, CDCl_3_, TMS): δ 20.02. FT-IR (cm^−1^): 3282 (w), 2928 (m), 1583 (w), 1562 (w), 1455 (w), 1431 (m), 1386 (w), 1332 (w), 1305 (w), 1260 (m), 1090 (m), 1025 (m), 969 (m), 938 (w), 913 (w), 859 (w), 827 (w), 795 (m), 738 (s), 691 (s). Anal. Calcd for C_30_H_31_N_2_P (1/2 H_2_O): C, 78.41; H,7.02; N, 6.10. Found: C, 78.10; H, 6.88; N, 5.95.

**N-(2-(diphenylphosphaneyl)ethyl)-5,6,7,8,9,10-hexahydrocycloocta[b]pyridin-10-amine (L8).** The synthesis of **L8** followed the same procedure as that for **L1**, except that 6,7,8,9-tetrahydrocycloocta[b]pyridin-10(5H)-one (1.35 g, 7.7 mmol) yielded the product as a yellow oily liquid (0.93 g, 31% yield). ^1^H NMR (400 MHz, CDCl_3_, TMS): δ 8.42 (d, *J* = 4.6 Hz, 1H), 7.41–7.30 (m, 5H), 7.28–7.21 (m, 6H), 7.07–7.01 (m, 1H), 4.11–4.05 (m, 1H), 2.81–2.50 (m, 4H), 2.36–2.17 (m, 2H), 2.04–1.97 (m, 2H), 1.68–1.52 (m, 2H), 1.43–1.23 (m, 4H). ^13^C NMR (100 MHz, CDCl_3_, TMS): δ 159.70, 146.28, 137.78, 137.69, 137.66, 137.56, 135.92, 135.48, 131.88, 131.69, 131.61, 131.43, 127.47, 127.34, 127.27, 120.62, 56.26, 44.07, 43.85, 37.68, 31.67, 31.53, 28.49, 28.37, 26.11, 23.40. ^31^P NMR (162 MHz, CDCl_3_, TMS): δ 20.51. FT-IR (cm^−1^): 3048 (w), 2923 (m), 1577 (m), 1476 (m), 1430 (s), 1368 (w), 1261 (m), 1184 (w), 1102 (m), 1068 (m), 1023 (m), 965 (w), 924 (w), 844 (w), 800 (m), 737 (s), 693 (s). Anal. Calcd for C_25_H_29_N_2_P(1/2 H_2_O): C, 75.54; H, 7.61; N, 7.05. Found: C, 75.40; H, 7.50; N, 6.94.

**N-(2-(diphenylphosphaneyl)ethyl)-6,7-dihydro-5H-cyclopenta[b]pyridin-7-aminozinc dichloride (Zn1). L1** (0.21 g, 0.60 mmol) and ZnCl_2_ (0.081 g, 0.60 mmol) were dissolved in 5 mL ethanol, respectively. Then, the ethanol solution of zinc chloride was added dropwise to the ligand solution and stirred for 12 h at room temperature under an atmosphere of nitrogen. The resulting precipitate was collected by direct filtration to yield **Zn1** as a green powder (0.19 g, 68% yield). ^1^H NMR (400 MHz, CDCl_3_, TMS): δ 8.80 (d, *J* = 5.0 Hz, 1H), 7.78–7.59 (m, 5H), 7.42–7.33 (m, 7H), 4.41–4.36 (m, 1H), 3.21–2.84 (m, 4H), 2.74–2.59 (m, 2H), 2.54–2.38 (m, 2H), 1.91–1.78 (m, 1H). ^13^C NMR (100 MHz, CDCl_3_, TMS): δ 162.00, 146.91, 137.48, 136.55, 133.32, 133.17, 133.10, 132.94, 130.24, 130.09, 129.07, 129.03, 128.99, 128.95, 124.66, 62.26, 28.11. ^31^P NMR (162 MHz, CDCl_3_, TMS): δ 25.86. FT-IR (cm^−1^): 3200 (m), 3073 (w), 2959 (w), 1606 (m), 1479 (w), 1436 (m), 1346 (w), 1321 (m), 1262 (m), 1204 (m), 1149 (w), 1096 (m), 1073 (w), 1034 (m), 1015 (m), 941 (m), 872 (w), 800 (m), 778 (w), 745 (s), 697 (s). Anal. Calcd for C_22_H_23_Cl_2_N_2_PZn (1/2H_2_O): C, 53.74; H, 4.92; N, 5.70. Found: C, 53.81; H, 4.82; N, 5.45.

**N-(2-(diphenylphosphaneyl)ethyl)-2-methyl-6,7-dihydro-5H-cyclopenta[b]pyridine-7-aminozinc dichloride (Zn2).** The synthesis of **Zn2** followed the same procedure as that for **Zn1**, except that **L2** (0.28 g, 0.77 mmol) was used to yield **Zn2** as a green powder (0.25 g, 65% yield). ^1^H NMR (400 MHz, CDCl_3_, TMS): δ 7.63 (d, *J* = 7.8 Hz, 1H), 7.57–7.50 (m, 2H), 7.44–7.37 (m, 5H), 7.35–7.28 (m, 3H), 7.16 (d, *J* = 7.8 Hz, 1H), 4.37–4.28 (m, 1H), 3.24 (s, 1H), 3.12–2.81 (m, 4H), 2.71 (s, 3H), 2.41–2.28 (m, 1H), 1.86–1.76 (m, 1H). ^13^C NMR (100 MHz, CDCl_3_, TMS): δ 160.85, 156.68, 136.97, 133.85, 133.42, 133.22, 132.54, 132.36, 129.45, 128.87, 128.80, 128.69, 128.62, 125.14, 63.15, 28.26, 23.09. ^31^P NMR (162 MHz, CDCl_3_, TMS): δ 22.19. FT-IR (cm^−1^): 3196 (m), 2955 (w), 1611 (m), 1587 (w), 1472 (m), 1434 (m), 1382 (w), 1348 (w), 1312 (w), 1265 (w), 1204 (w), 1144 (m), 1096 (m), 1074 (m), 1015 (m), 973 (w), 942 (w), 859 (m), 834 (m), 798 (m), 743 (s), 698 (s). Anal. Calcd for C_23_H_25_Cl_2_N_2_PZn: C, 55.62; H, 5.07; N, 5.64. Found: C, 55.29; H, 5.08; N, 5.42.

**N-(2-(diphenylphosphaneyl)ethyl)-2-phenyl-6,7-dihydro-5H-cyclopenta[b]pyridin-7-aminozinc chloride (Zn3).** The synthesis of **Zn3** followed the same procedure as that for **Zn1**, except that **L3** (0.21 g, 0.49 mmol) was used to yield **Zn3** as a green powder (0.20 g, 73% yield). ^1^H NMR (400 MHz, CDCl_3_, TMS): δ 7.91–7.86 (m, 2H), 7.73 (d, *J* = 7.9 Hz, 1H), 7.60–7.49 (m, 6H), 7.44–7.35 (m, 5H), 7.33–7.28 (m, 3H), 4.48–4.37 (m, 1H), 3.24 (s, 1H), 3.12–2.87 (m, 4H), 2.78–2.67 (m, 2H), 2.25–2.14 (m, 1H), 1.89–1.77 (m, 1H). ^13^C NMR (100 MHz, CDCl_3_, TMS): δ 161.93, 157.37, 137.20, 136.73, 135.32, 133.38, 133.19, 132.62, 132.44, 130.40, 129.38, 128.84, 128.76, 128.66, 128.59, 128.08, 123.99, 63.15, 45.99, 32.64, 28.31, 27.65. ^31^P NMR (162 MHz, CDCl_3_, TMS): δ 22.20. FT-IR (cm^−1^): 3193 (m), 1597 (w), 1576 (w), 1457 (m), 1433 (m), 1339 (w), 1309 (w), 1230 (m), 1206 (w), 1157 (m), 1100 (m), 1072 (m), 1043 (w), 1009 (m), 971 (m), 945 (m), 842 (m), 791 (w), 736 (s), 694 (s). Anal. Calcd for C_28_H_27_Cl_2_N_2_PZn: C, 60.18; H, 4.87; N, 5.01. Found: C, 59.99; H, 4.84; N, 4.86.

**N-(2-(diphenylphosphaneyl)ethyl)-5,6,7,8-tetrahydroquinolin-8-aminozinc dichloride (Zn4)** [51]. The synthesis of **Zn4** followed the same procedure as that for **Zn1**, except that **L4** (0.37 g, 1.03 mmol) was used to yield **Zn4** as a white powder (0.38 g, 74% yield).

**N-(2-(diphenylphosphaneyl)ethyl)-2-phenyl-5,6,7,8-tetra-hydroquinolin-8-aminozinc dichloride (Zn5)** [40]**.** The synthesis of **Zn5** followed the same procedure as that for **Zn1**, except that **L5** (0.28 g, 0.64 mmol) was used to yield **Zn5** as a white powder (0.32 g, 87% yield).

**N-(2-(diphenylphosphaneyl)ethyl)-6,7,8,9-tetrahydro-5H-cyclohepta[b]pyridin-9-aminozinc dichloride (Zn6).** The synthesis of **Zn6** followed the same procedure as that for **Zn1**, except that **L6** (0.39 g, 1.0 mmol) was used to yield **Zn6** as a white powder (0.42 g, 78% yield). ^1^H NMR (400 MHz, CDCl_3_, TMS): δ 8.56 (d, *J* = 4.3 Hz, 1H), 7.68 (d, *J* = 7.4 Hz, 1H), 7.59–7.47 (m, 4H), 7.40–7.31 (m, 7H), 4.06 (d, *J* = 8.1 Hz, 1H), 3.13–2.66 (m, 6H), 2.53–2.48 (m, 1H), 2.07–1.73 (m, 5H). ^13^C NMR (100 MHz, CDCl_3_, TMS): δ 156.82, 144.32, 139.75, 137.35, 132.14, 131.95, 131.90, 131.72, 128.42, 128.35, 127.87, 127.85, 127.80, 127.77, 123.37, 32.33, 28.33, 26.29, 24.67. ^31^P NMR (162 MHz, CDCl_3_, TMS): δ 23.01. FT-IR (cm^−1^): 3201 (m), 3070 (w), 2951 (w), 1591 (m), 1478 (m), 1450 (m), 1431 (s), 1357 (w), 1333 (w), 1261 (m), 1204 (m), 1165 (w), 1094 (m), 1028 (m), 944 (m), 800 (m), 769 (w), 740 (s), 695 (s). Anal. Calcd for C_24_H_27_Cl_2_N_2_PZn (1/2H_2_O): C, 55.46; H, 5.43; N, 5.39. Found: C, 55.22; H, 5.45; N, 5.01.

**N-(2-(diphenylphosphaneyl)ethyl)-2-phenyl-6,7,8,9-tetrahydro-5H-cyclohepta[b]pyridin-9-aminozinc dichloride (Zn7).** The synthesis of **Zn7** followed the same procedure as that for **Zn1**, except that **L7** (0.22 g, 0.46 mmol) was used to yield **Zn7** as a white powder (0.21 g, 78% yield). ^1^H NMR (400 MHz, CDCl_3_, TMS): δ 7.83 (d, *J* = 6.2 Hz, 2H), 7.73 (d, *J* = 6.9 Hz, 1H), 7.58–7.45 (m, 8H), 7.39–7.31 (m, 6H), 4.18 (s, 1H), 3.27 (s, 1H), 2.94–2.81 (m, 4H), 2.77–2.64 (m, 2H), 2.56–2.42 (m, 1H), 2.18–1.92 (m, 4H), 1.91–1.79 (m, 1H). ^13^C NMR (100 MHz, CDCl_3_, TMS): δ 161.54, 153.16, 139.48, 138.75, 138.68, 138.63, 138.55, 137.76, 135.35, 132.80, 132.78, 132.62, 132.59, 128.60, 128.48, 128.45, 128.42, 128.35, 126.71, 117.91, 63.07, 45.50, 45.28, 34.29, 33.90, 29.46, 29.35, 29.20, 27.41. ^31^P NMR (162 MHz, CDCl_3_, TMS): δ 20.02. FT-IR (cm^−1^): 3282 (m), 3057 (w), 2937 (m), 1588 (m), 1566 (m), 1458 (m), 1433 (m), 1388 (m), 1362 (m), 1305 (w), 1234 (m), 1203 (m), 1073 (m), 1045 (m), 987 (m), 931 (m), 910 (m), 848 (m), 749 (s), 700 (s). Anal. Calcd for C_30_H_31_Cl_2_N_2_PZn: C, 61.40; H, 5.32; N, 4.77. Found: C, 61.28; H, 5.33; N, 4.58.

**N-(2-(diphenylphosphaneyl)ethyl)-5,6,7,8,9,10-hexahydrocycloocta[b]pyridin-10-aminozinc chloride (Zn8).** The synthesis of **Zn8** followed the same procedure as that for **Zn1**, except that **L8** (0.19 g, 0.49 mmol) was used to yield **Zn8** as a white powder (0.20 g, 78% yield). ^1^H NMR (400 MHz, CDCl_3_, TMS): δ 8.66 (d, *J* = 4.4 Hz, 1H), 7.70 (d, *J* = 7.5 Hz, 1H), 7.54–7.39 (m, 6H), 7.37–7.32 (m, 5H), 4.38–4.27 (m, 1H), 3.29 (s, 1H), 3.03–2.88 (m, 1H), 2.85–2.60 (m, 4H), 2.55–2.42 (m, 1H), 2.28–2.17 (m, 1H), 2.08–1.92 (m, 2H), 1.66–1.51 (m, 3H), 1.43–1.33 (m, 2H). ^13^C NMR (100 MHz, CDCl_3_, TMS): δ 155.60, 146.90, 141.03, 138.84, 135.83, 132.95, 132.77, 129.37, 129.26, 128.88, 128.81, 128.74, 124.79, 60.69, 48.20, 37.26, 32.00, 26.32, 23.92. ^31^P NMR (162 MHz, CDCl_3_, TMS): δ 22.29. FT-IR (cm^−1^): 3235. (m), 3059.60 (w), 2925 (m), 1598 (m), 1452 (w), 1436 (m), 1357 (m), 1260 (m), 1197 (m), 1094 (m), 1056 (m), 1015 (s), 948 (m), 868 (w), 801 (m), 745 (w), 736 (s), 696 (s). Anal. Calcd for C_25_H_29_Cl_2_N_2_PZn (1/2 EtOH): C, 57.01; H, 5.89; N, 5.11 Found: C, 56.70; H, 5.60; N, 5.05.

#### 3.2.2. General Procedure for Ring-Opening Polymerization of L-lactide or rac-LA

A typical polymerization procedure is outlined as follows using **Zn6** as the representative precatalyst. In a Schlenk flask, **Zn6** (0.0051 g, 0.010 mmol) and LiN(SiMe_3_)_2_ (0.02 mmol, 2 equiv) were dissolved in 1 mL toluene and the mixture was stirred at room temperature for 30 min under a nitrogen atmosphere. Then, the **Zn6**/LiN(SiMe_3_)_2_ was immediately injected into the monomer (*L*-LA, 0.36 g, 2.5 mmol) to react for the designated time at a specified temperature. After the reaction, the resulting viscous solution was transferred to a flask containing 100 mL cold methanol and stirred. The ring-opening polymerization procedure of the rac-lactide is similar to that of the L-lactide above.

#### 3.2.3. X-ray Crystallographic Analyses

Single-crystal X-ray diffraction data for **Zn2**, **Zn3**, **Zn7** and **Zn8** were conducted on an XtaLAB Synergy-R HyPix diffractometer with graphite-monochromated Cu-Kα radiation (λ = 1.54184 (Å)) at 169.98(10) K. The cell parameters were obtained through global optimization of the positions of all the reflected signals collected. Intensities were corrected for Lorentz and polarization effects and empirical absorption. The structures were solved by direct methods and refined by full-matrix least squares on *F*^2^. All hydrogen atoms were placed in calculated positions. Structure solution and refinement were performed using the SHELXTL-97 package [72,73,74]. Crystal data and structure refinements for **Zn2**, **Zn3**, **Zn7** and **Zn8** are summarized in Table 4.

## 4. Conclusions

In summary, the finely tuned ring sizes of the cycloalkyl-fused pyridine-bearing pendant N-diphenylphosphinoethyl groups are developed as ligands **L1**–**L8** for their zinc chloride complexes **Zn1**–**Zn8**. The catalytic systems, **Zn1**–**Zn8**/2Li(NSiMe_3_)_2_, display good activities toward the ROP of *L*-LA and *rac*-LA in situ without BnOH. In general, the cyclohexyl- or cyclooctyl-fused pyridyl–zinc catalysts demonstrate competitively higher activities. For *L*-LA, 92% monomer conversion was achieved with the [*L*-LA]: [Zn] molar ratio of 500:1 in only 5 min and at 80 °C. By comparison, the activity for the ROP of rac-LA were generally observed to be much lower than that seen for *L*-LA, in which the highest monomer conversion could only reach 74% under the same polymerization conditions and all of the obtained poly(*rac*-LA) product was amorphous. The microstructures of PLAs indicate the linear structures capped with a CH_3_O- end group were the major and the zwitterionic species was proposed as the intermediate in the polymerization.

## Figures and Tables

**Table 1:** Selected bond lengths and angles for **Zn2**, **Zn3**, **Zn7** and **Zn8**.

	Zn2	Zn3	Zn7	Zn8
Bond lengths (Å)				
Zn1–Cl1	2.2018(5)	2.2220(6)	2.230(2)	2.2214(4)
Zn1–Cl2	2.2287(5)	2.2053(6)	2.220(2)	2.2081(4)
Zn1–N1	2.0777(15)	2.1162(16)	2.085(7)	2.0442(13)
Zn1–N2	2.1091(15)	2.0846(17)	2.080(7)	2.0920(12)
Bond angles (°)				
Cl2–Zn1–Cl1	115.75(2)	118.29(2)	116.84(10)	117.623(18)
N1–Zn1–Cl1	114.14(5)	106.87(5)	111.9(2)	117.20(4)
N1–Zn1–Cl2	111.37(5)	118.83(5)	118.9(2)	105.86(4)
N1–Zn1–N2	85.08(6)	85.86(7)	82.4(3)	82.74(5)
N2–Zn1–Cl1	113.51(5)	107.26(5)	112.7(2)	109.14(4)
N2–Zn1–Cl2	113.23(5)	114.83(5)	109.0(2)	119.54(4)

**Table 2:** Ring-opening polymerization of *L*-LA by **Zn1**–**Zn8**/2LiN(SiMe_3_)_2_^a^.

Run	Cat	*L*-LA:Zn	Solvent	T/°C	t/min	Conv./% ^b^	TOF/h^−1^	M_n(calcd)_^c^	M_n_^d^	M_w_/M_n_^d^
1	**Zn6**	250:1	toluene	30	10	36	540	1.30	3.99	1.74
2 ^e^	**Zn6**	250:1	toluene	30	10	11	165	0.39	0.59	1.17
3 ^f^	**Zn6**	250:1	toluene	30	10	8	120	0.28	0.51	1.15
4	**Zn6**	250:1	toluene	30	20	48	360	1.73	4.19	1.62
5	**Zn6**	250:1	toluene	30	40	65	244	2.34	3.06	1.72
6	**Zn6**	250:1	toluene	30	60	86	215	3.10	1.65	2.03
7	**Zn6**	250:1	toluene	50	10	68	1020	2.45	2.72	1.87
8	**Zn6**	250:1	toluene	60	10	85	1275	3.06	2.83	2.06
9	**Zn6**	250:1	toluene	70	10	96	1440	3.46	2.77	1.87
10	**Zn6**	250:1	toluene	80	10	100	1500	3.60	2.66	2.08
11	**Zn6**	250:1	CH_2_Cl_2_	30	10	0	0	–	–	–
12	**Zn6**	250:1	THF	60	10	92	1380	3.31	1.58	2.12
13	**Zn6**	250:1	hexane	60	10	8	120	0.29	2.52	1.97
14	**Zn6**	500:1	toluene	80	10	96	2880	6.91	2.49	2.13
15	**Zn6**	1000:1	toluene	80	10	89	5340	12.81	2.61	2.13
16 ^g^	**Zn6**	1000:1	toluene	80	10	79	4740	11.38	2.38	1.79
17 ^h^	**Zn6**	1000:1	toluene	80	10	65	3900	9.36	2.02	2.33
18	**Zn6**	500:1	toluene	80	5	63	3780	4.54	1.86	1.97
19	**Zn1**	500:1	toluene	80	5	75	4500	5.40	1.96	1.90
20	**Zn2**	500:1	toluene	80	5	70	4200	5.04	1.68	2.04
21	**Zn3**	500:1	toluene	80	5	44	2640	3.17	2.68	2.01
22	**Zn4**	500:1	toluene	80	5	92	5520	6.62	3.90	1.75
23	**Zn5**	500:1	toluene	80	5	53	3180	3.82	1.51	2.17
24	**Zn7**	500:1	toluene	80	5	36	2160	2.59	1.92	1.99
25	**Zn8**	500:1	toluene	80	5	92	5520	6.62	1.84	2.27

^a^ Reaction conditions: 1.0 mL toluene, 10 μmol [Zn] + 20 μmol LiN(SiMe_3_)_2_; ^b^ determined by ^1^H NMR spectroscopy; ^c^ 10^4^ g/mol, M_n(calcd)_ = molar ratio of [L-LA]/[Zn] × 144 × conv.%; ^d^ 10^4^ g/mol, GPC data were recorded from THF vs. polystyrene standards using a correcting factor of 0.58 [58]; ^e^ 1 equiv. BnOH; ^f^ 3 equiv. BnOH; ^g^ 3 mL toluene; ^h^ 5 mL toluene.

**Table 3:** Ring-opening polymerization of *rac*-LA by **Zn1**–**Zn8**/2LiN(SiMe_3_)_2_^a^.

Run	Cat	rac-LA: Zn	T/°C	t/min	Conv./% ^b^	TOF/h^−1^
1	**Zn1**	500:1	80	5	59	3540
2	**Zn2**	500:1	80	5	53	3180
3	**Zn3**	500:1	80	5	52	3120
4	**Zn4**	500:1	80	5	71	4260
5	**Zn5**	500:1	80	5	53	3180
6	**Zn6**	500:1	80	5	46	2760
7	**Zn7**	500:1	80	5	18	1080
8	**Zn8**	500:1	80	5	74	4440

^a^ Reaction conditions: 1.0 mL toluene, 10 μmol [Zn] + 20 μmol LiN(SiMe_3_)_2_; ^b^ determined by ^1^H NMR spectroscopy.

**Table 4:** Crystal data and structure refinements for **Zn2**, **Zn3**, **Zn7** and **Zn8**.

	Zn2	Zn3	Zn7	Zn8
empirical formula	C_23_H_25_Cl_2_N_2_PZn	C_28_H_27_Cl_2_N_2_PZn	C_30_H_31_Cl_2_N_2_PZn	C_25_H_29_Cl_2_N_2_PZn
formula weight	496.69	558.75	586.81	524.74
temperature/K	170.00(10)	169.98(10)	169.98(10)	169.98(10)
crystal system	triclinic	triclinic	monoclinic	triclinic
space group	P-1	P-1	P2_1_/c	P-1
a/Å	9.2670(2)	8.8906(3)	12.9499(5)	9.5988(3)
b/Å	10.2665(3)	10.7637(3)	16.8402(6)	10.9347(4)
c/Å	12.7846(3)	15.1833(5)	13.0719(5)	13.0072(4)
α/˚	84.143(2)	108.512(3)	90	84.306(3)
β/˚	75.169(2)	105.259(3)	106.844(4)	75.326(3)
γ/˚	87.433(2)	94.871(2)	90	74.958(3)
volume/Å^3^	1169.44(5)	1306.49(8)	2728.40(19)	1274.64(8)
Z	2	2	4	2
ρcalc/g cm^3^	1.411	1.420	1.429	1.367
μ/mm^−1^	4.289	3.908	3.770	3.964
F (000)	512.0	576.0	1216.0	544.0
crystal size/mm^3^	0.25 × 0.25 × 0.2	0.3 × 0.3 × 0.25	0.15 × 0.1 × 0.02	0.33 × 0.3 × 0.28
radiation	CuKα (λ = 1.54184)	CuKα (λ = 1.54184)	CuKα (λ = 1.54184)	CuKα (λ = 1.54184)
2θ range for data collection/°	7.184 to 154.478	6.45 to 153.556	7.132 to 154.446	8.378 to 154.348
index ranges	−11 ≤ h ≤ 11, −12 ≤ k ≤ 12, −16 ≤ l ≤ 14	−11 ≤ h ≤ 11, −13 ≤ k ≤ 13, −18 ≤ l ≤ 19	−16 ≤ h ≤ 16, −20 ≤ k ≤ 21, −16 ≤ l ≤ 16	−12 ≤ h ≤ 12, −13 ≤ k ≤ 13, −16 ≤ l ≤ 14
reflections collected	13,881	15,346	19,546	15,774
independent reflections	4716 [R_int_ = 0.0236, R_sigma_ = 0.0229]	5274 [R_int_ = 0.0245, R_sigma_ = 0.0314]	5574 [R_int_ = 0.0669, R_sigma_ = 0.0560]	5156 [R_int_ = 0.0218, R_sigma_ = 0.0196]
data/restraints/parameters	4716/0/263	5274/0/307	5574/6/325	5156/0/281
goodness-of-fit on *F*^2^	1.050	1.032	1.100	1.024
final R indices [*I* ≥ 2σ (*I*)]	R_1_ = 0.0319, wR_2_ = 0.0838	R_1_ = 0.0367, wR_2_ = 0.0969	R_1_ = 0.1142, wR_2_ = 0.3324	R_1_ = 0.0272, wR_2_ = 0.0722
final R indices [all data]	R_1_ = 0.0333, wR_2_ = 0.0849	R_1_ = 0.0388, wR_2_ = 0.0989	R_1_ = 0.1221, wR_2_ = 0.3364	R_1_ = 0.0281, wR_2_ = 0.0730
largest diff. peak/hole/e Å^−3^	1.13/−0.43	1.53/−0.39	2.67/−0.93	1.04/−0.35

## Data Availability

The original contributions presented in the study are included in the article/Supplementary Materials, further inquiries can be directed to the corresponding authors.

## Supplementary Materials

The following supporting information can be downloaded at https://www.mdpi.com/article/10.3390/molecules29174150/s1. Figures S1–S36: ^1^H/^13^C/^3l^P NMR spectra of ligand and zinc complexes. Figure S37: Kinetics plot of polymerization of *rac*-LA by **Zn6/**2LiN(SiMe_3_)_2_. Figures S38 and S39: MALDI-TOF and ^1^H NMR spectra of PLAs. Figure S40: Decoupling ^1^H NMR spectrum of the poly(*rac*-LA) obtained using **Zn1-Zn8**/2LiN(SiMe_3_)_2_. CCDC 2330187-2330190 for **Zn2**, **Zn3**, **Zn7** and **Zn8**. For crystallographic data in CIF or another electronic format, see Supplementary Materials.
