# Supplementary material for: Intensive Cycloalkyl-Fused Pyridines for Aminopyridyl–Zinc–Heteroimidazoles Achieving High Efficiency toward the Ring-Opening Polymerization of Lactides
Source: Molecules. 2024 Aug 31;29(17):4150. doi: 10.3390/molecules29174150 (PMC11397438; doi:10.3390/molecules29174150)
Supplement: Supplementary file 1 [file molecules-29-04150-s001.zip › molecules-3154353-supplementary.pdf]

Supporting information

of

Intensive cycloalkyl-fused pyridines for the aminopyridyl zinc-heteroimidazoles achieving highly efficiency toward the ring-opening polymerization of lactides

Yun Wang,[a,b] Wenjuan Zhang,*[a] Pengjiang Zhu,[a,b] Wei You,[b] Xiaopan Xue,[a,b] Rui Wang,[a] Yanping Ma,[b] Wen-Hua Sun*[b]

[a] Beijing Key Laboratory of Clothing Materials R&D and Assessment, Beijing Engineering Research Center of Textile Nanofiber, School of Materials Science and Engineering, Beijing Institute of Fashion Technology, Beijing 100029, China. E-mail: zhangwj@bift.edu.cn.

[b] Key Laboratory of Engineering Plastics and Beijing National Laboratory for Molecular Science, Institute of Chemistry, Chinese Academy of Sciences, Beijing 100190, China. E-mail: whsun@iccas.ac.cn

Table of contents

		Page
1	**Figure S1-S3** $^{1}H/^{13}C/^{31}P$ NMR spectrum of **L1**	S2-S3
2	**Figure S4-S6** $^{1}H/^{13}C/^{31}P$ NMR spectrum of **L2**	S3-S4
3	**Figure S7-S9** $^{1}H/^{13}C/^{31}P$ NMR spectrum of **L3**	S5-S6
4	**Figure S10-S12** $^{1}H/^{13}C/^{31}P$ NMR spectrum of **L6**	S6-S7
5	**Figure S13-S15** $^{1}H/^{13}C/^{31}P$ NMR spectrum of **L7**	S8-S9
6	**Figure S16-S18** $^{1}H/^{13}C/^{31}P$ NMR spectrum of **L8**	S9-S10
7	**Figure S19-S21** $^{1}H/^{13}C/^{31}P$ NMR spectrum of **Zn1**	S11-S12
8	**Figure S22-S24** $^{1}H/^{13}C/^{31}P$ NMR spectrum of **Zn2**	S12-S13
9	**Figure S25-S27** $^{1}H/^{13}C/^{31}P$ NMR spectrum of **Zn3**	S14-S15
10	**Figure S28-S30** $^{1}H/^{13}C/^{31}P$ NMR spectrum of **Zn6**	S15-S16
11	**Figure S31-S33** $^{1}H/^{13}C/^{31}P$ NMR spectrum of **Zn7**	S17-S18
12	**Figure S34-S36** $^{1}H/^{13}C/^{31}P$ NMR spectrum of **Zn8**	S18-S19
13	**Table S1** Comparison of the ^{31}P NMR chemical shifts for ligands with those in zinc complexes	S20
14	**Figure S37** Kinetics plot of polymerization of *rac*-LA by **Zn6**/2LiN(SiMe$_3$)$_2$ (LA:Zn = 250:1 or 250:0.5, 30°C, toluene)	S20
15	**Figure S38** MAIDI-TOF spectrum of the poly(*rac*-LA) obtained using **Zn6**/2LiN(SiMe$_3$)$_2$ (run 1, Table 3)	S21
16	**Figure S39** ^{1}H NMR spectrum of the poly(*rac*-LA) obtained using **Zn6**/2LiN(SiMe$_3$)$_2$ (run 1, Table 3)	S21
17	**Figure S40** Decoupling ^{1}H NMR spectrum of the poly(*rac*-LA) obtained using **Zn1-Zn8**/2LiN(SiMe$_3$)$_2$ (Table 3)	S22

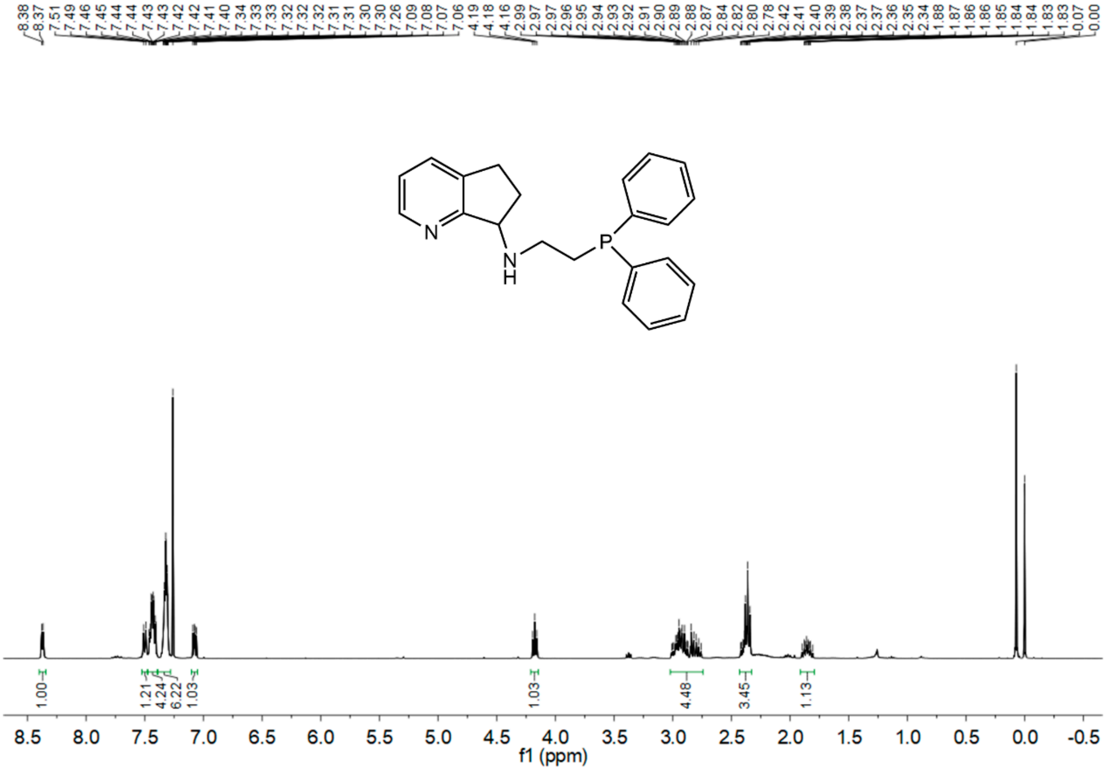

Figure S1 ^{1}H NMR ($CDCl_3$, 25°C) spectrum of **L1**

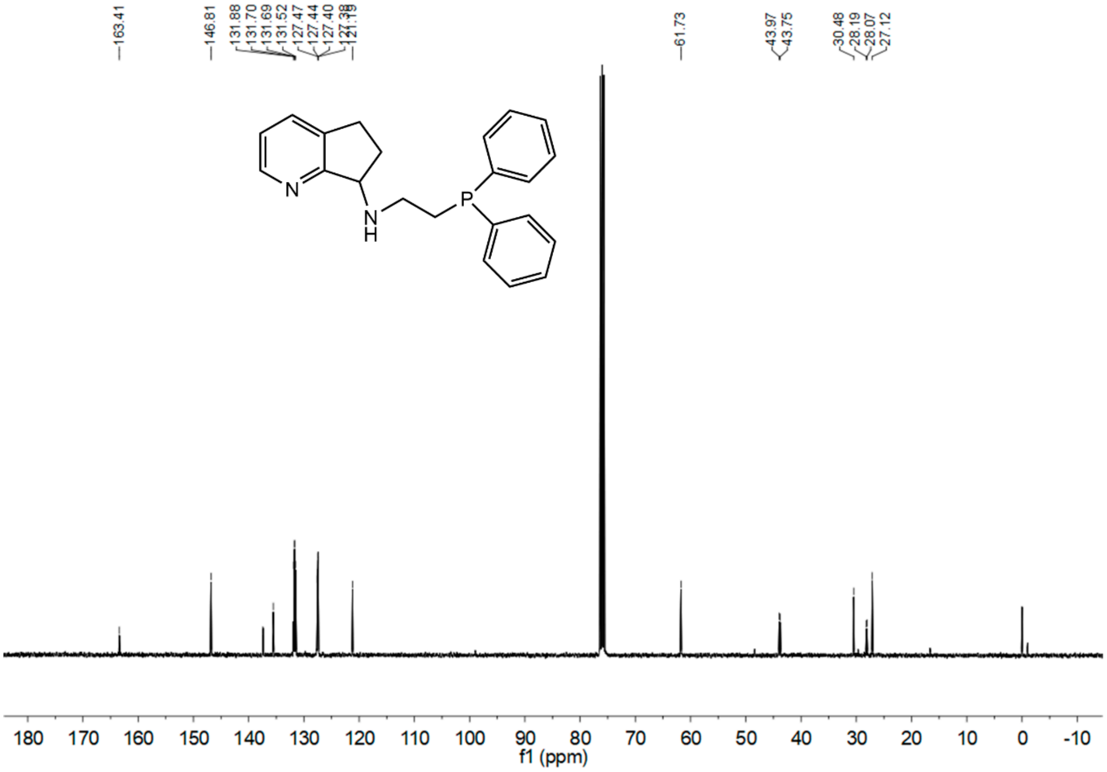

Figure S2 ^{13}C NMR ($CDCl_3$, 25°C) spectrum of **L1**

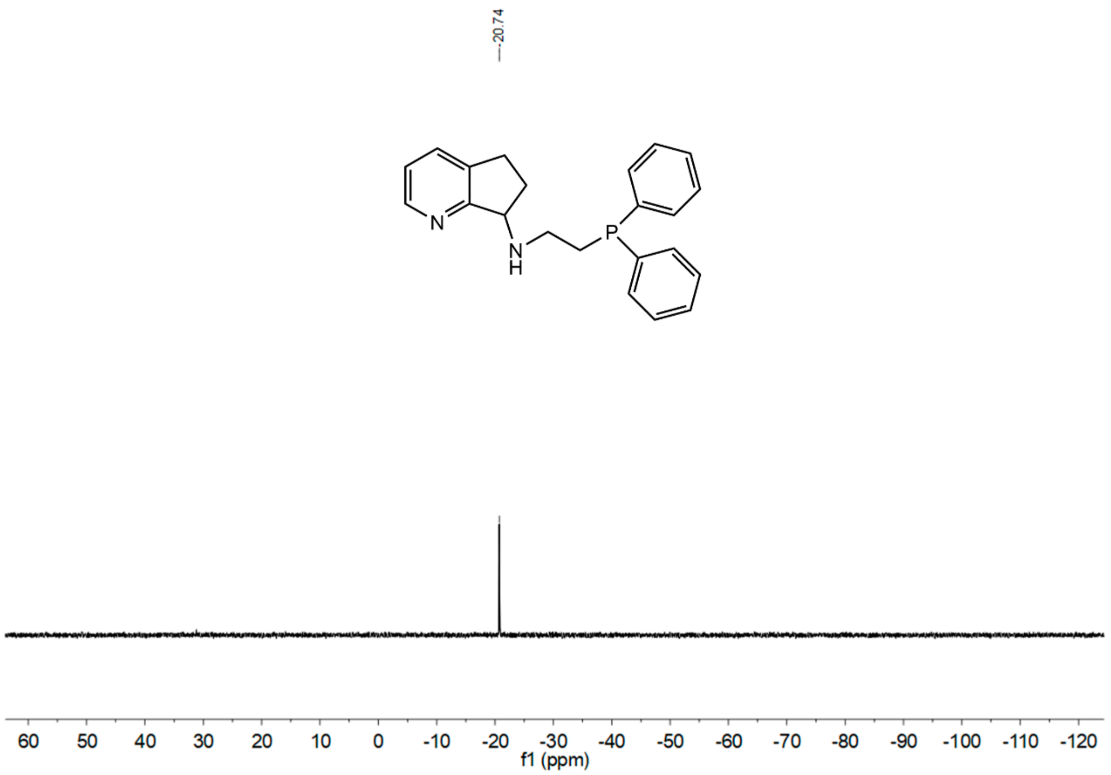

Figure S3 ^{31}P NMR ($CDCl_3$, 25°C) spectrum of **L1**

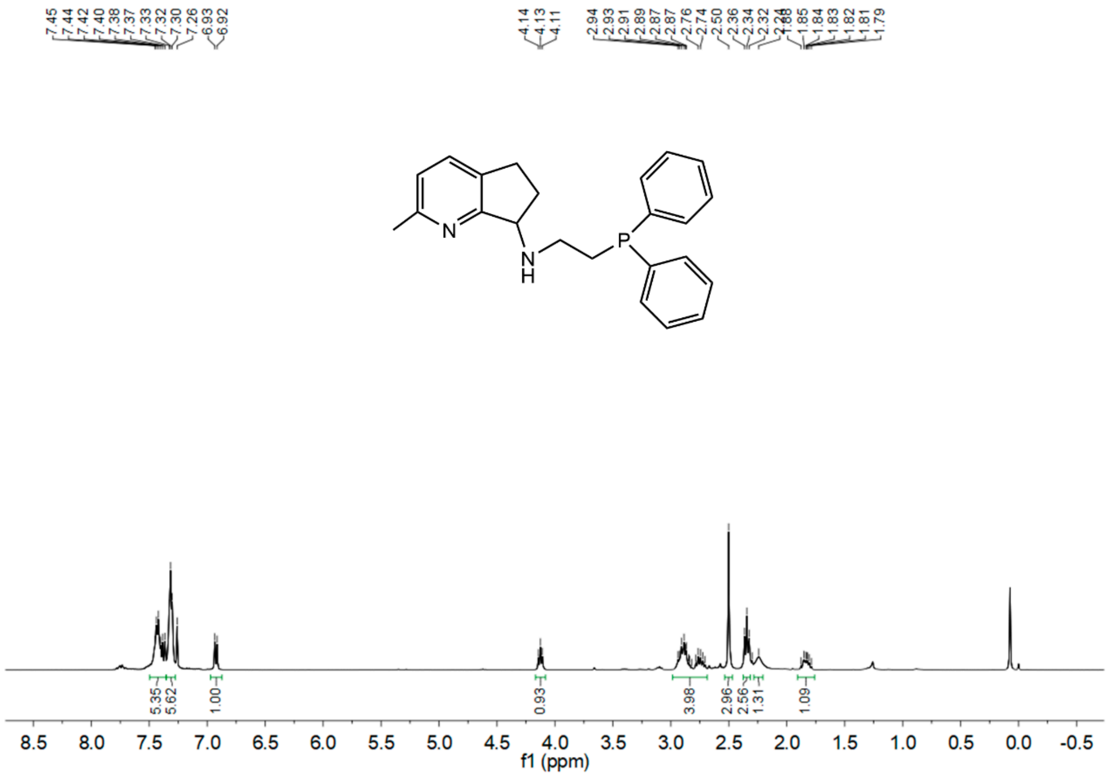

Figure S4 ^{1}H NMR ($CDCl_3$, 25°C) spectrum of **L2**

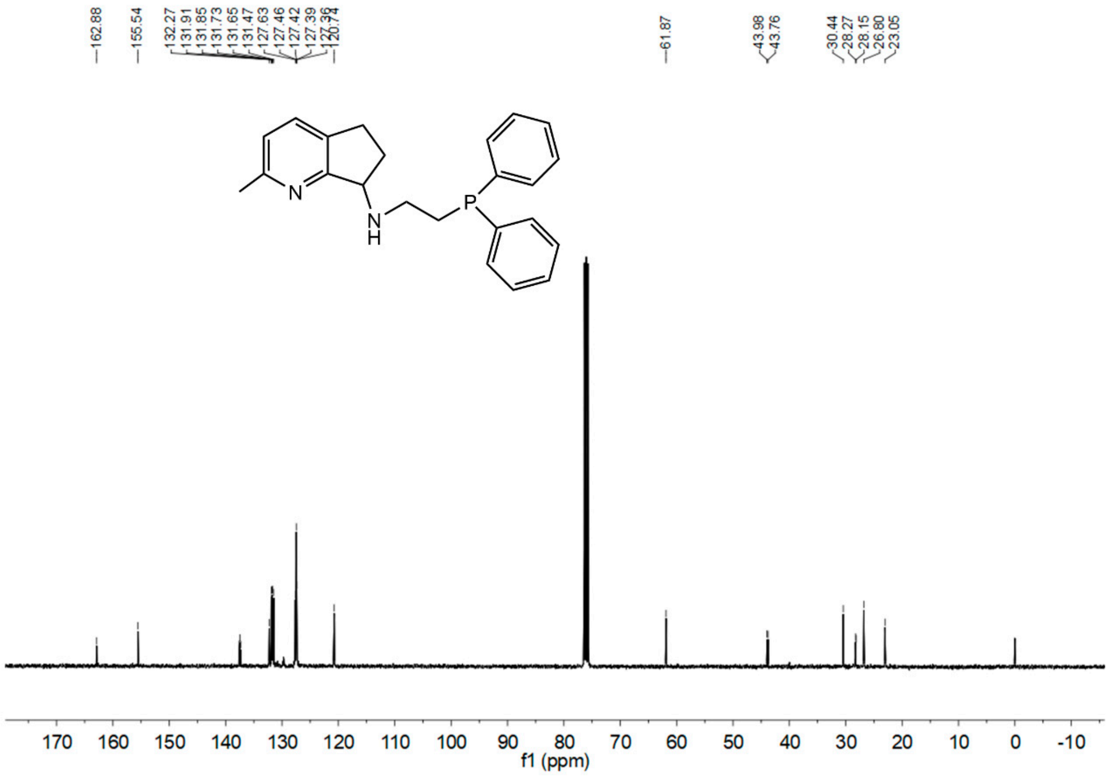

Figure S5 ^{13}C NMR ($CDCl_3$, 25°C) spectrum of **L2**

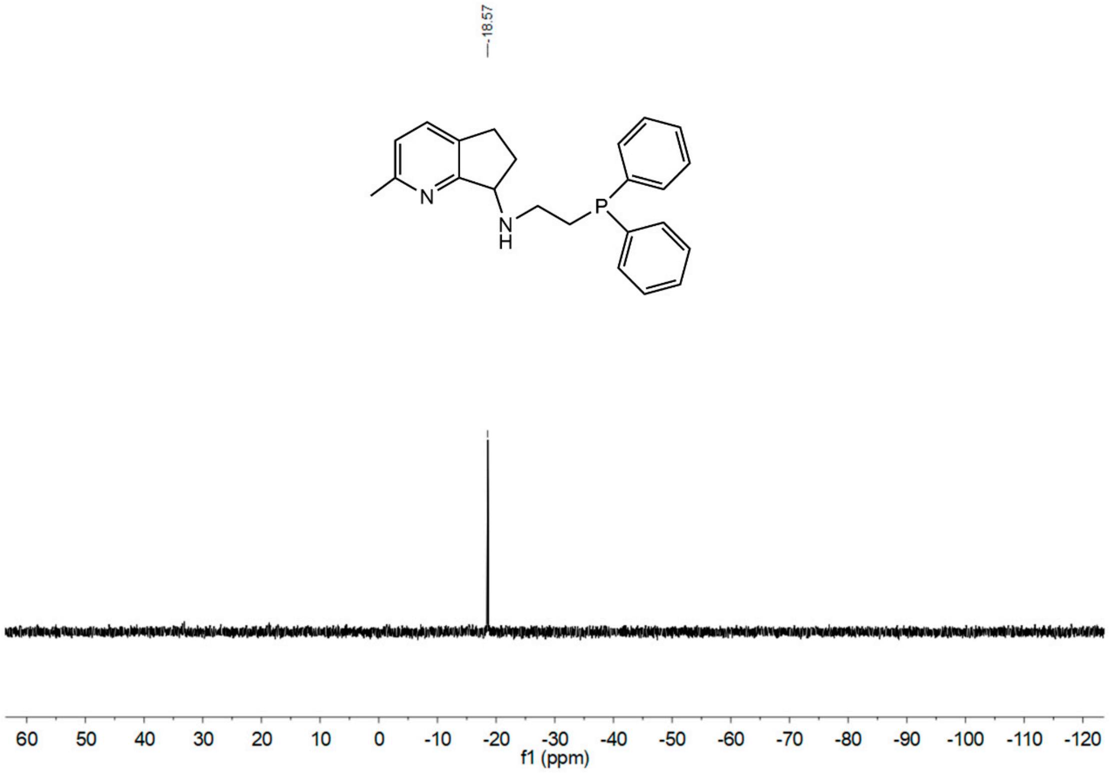

Figure S6 ^{31}P NMR ($CDCl_3$, 25°C) spectrum of **L2**

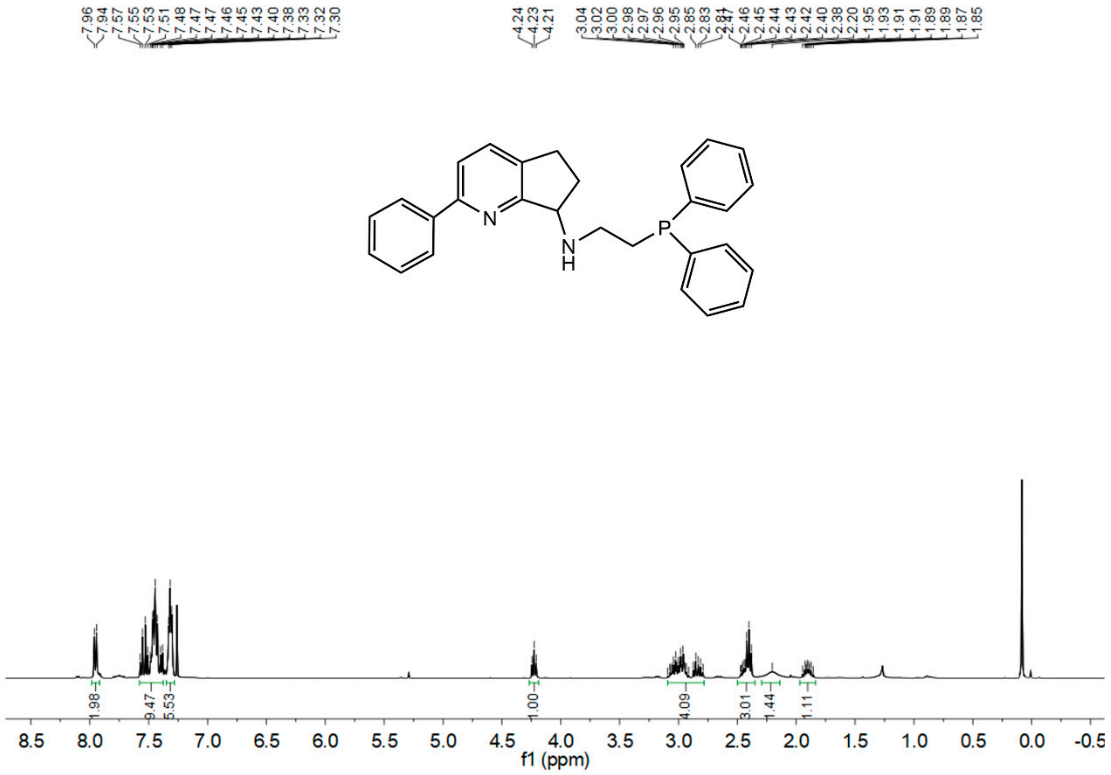

Figure S7 ^{1}H NMR (CDCl₃, 25°C) spectrum of **L3**

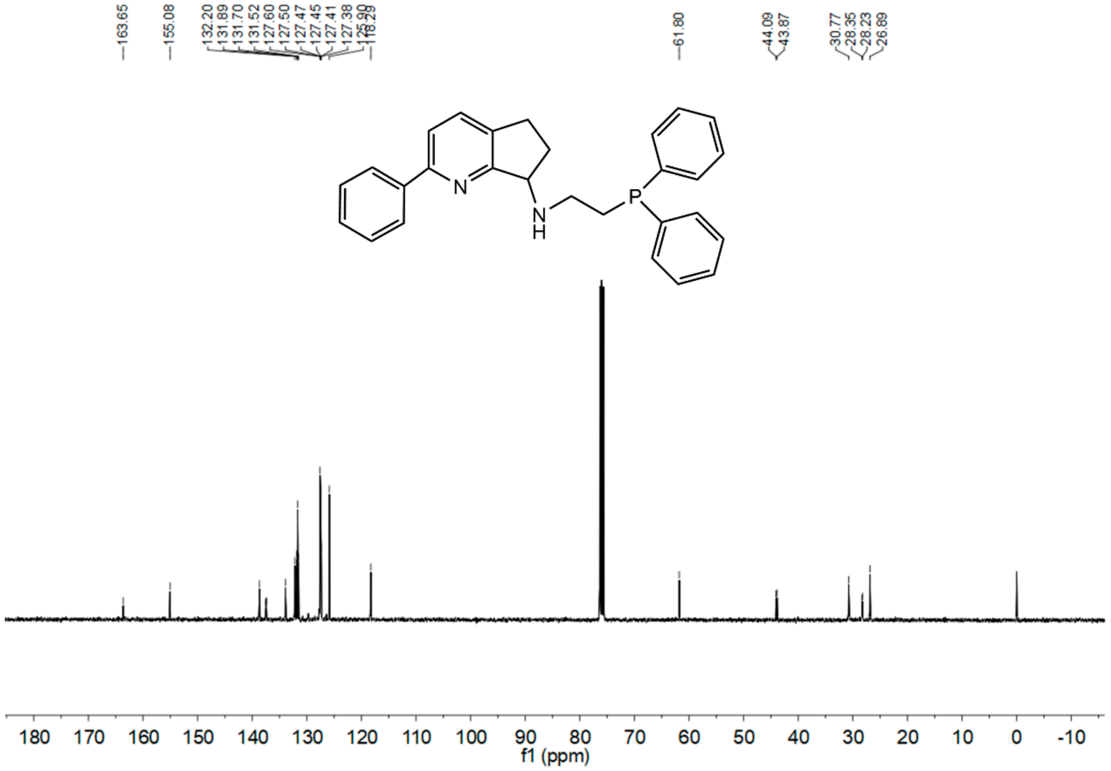

Figure S8 ^{13}C NMR (CDCl₃, 25°C) spectrum of **L3**

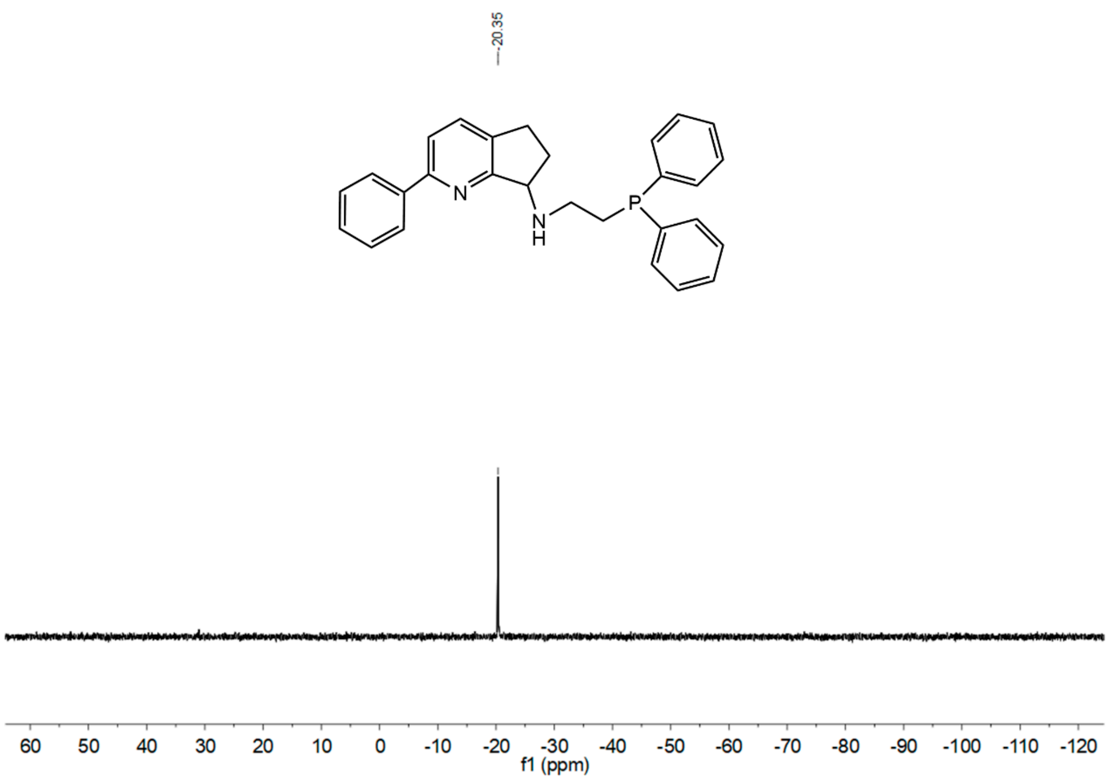

Figure S9 ^{31}P NMR ($CDCl_3$, 25°C) spectrum of **L3**

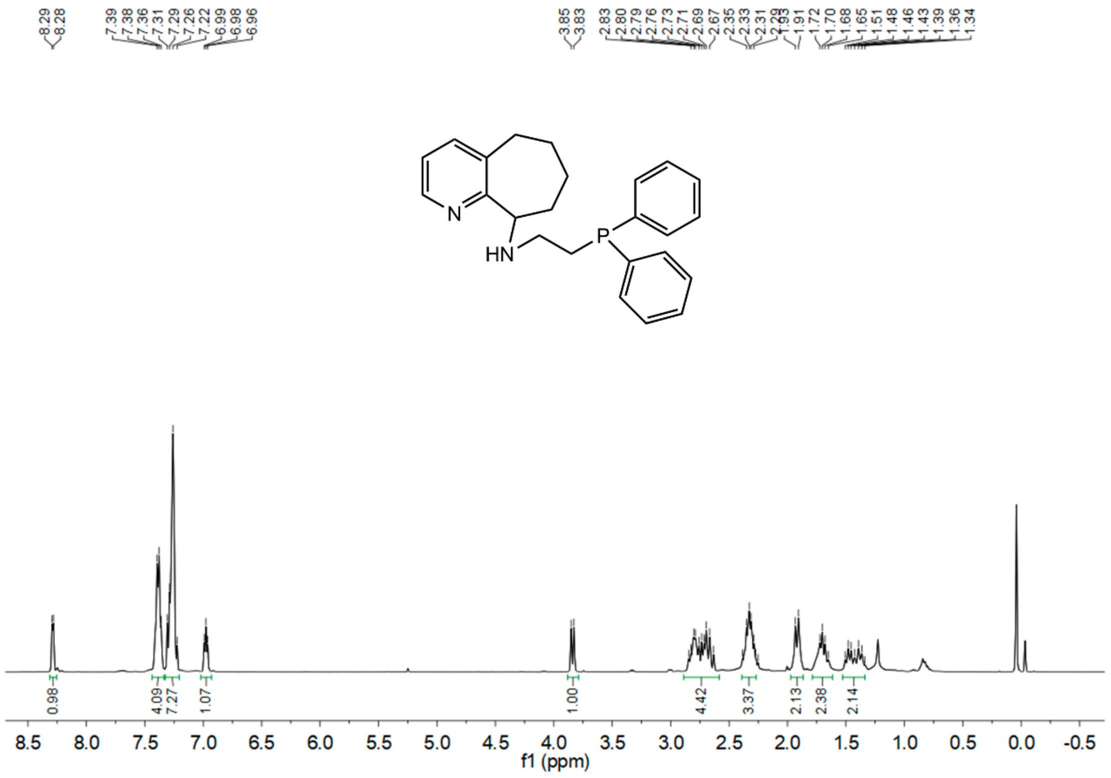

Figure S10 ^{1}H NMR ($CDCl_3$, 25°C) spectrum of **L6**

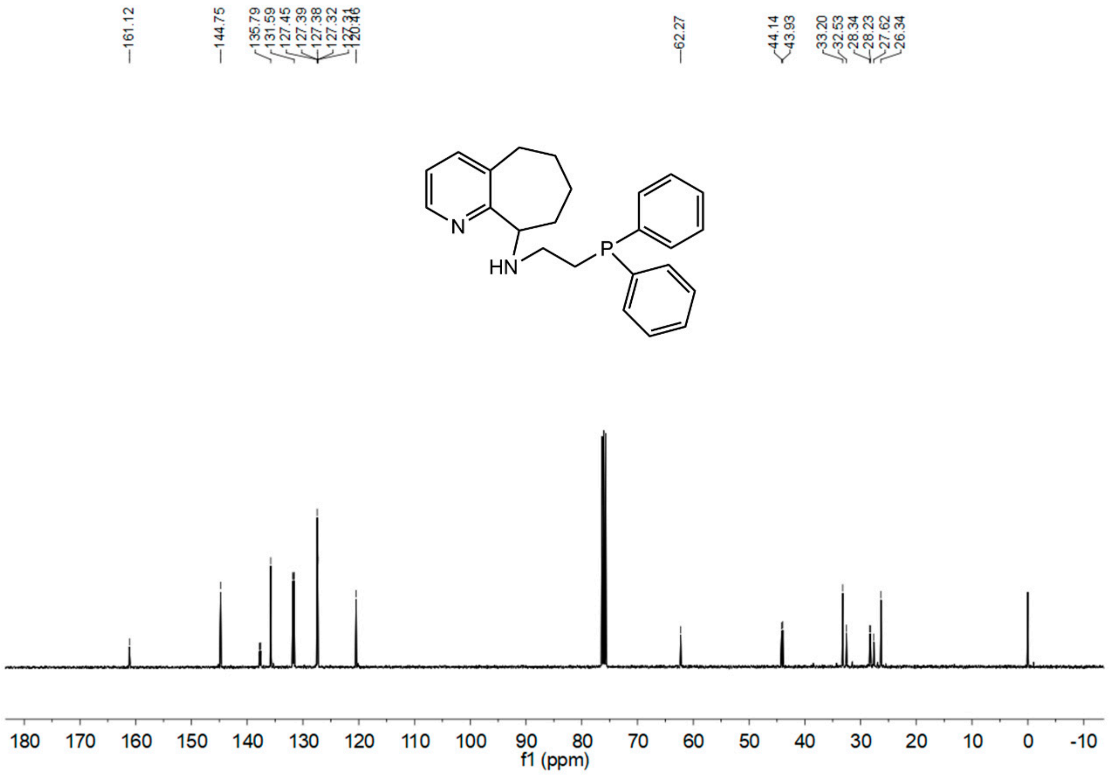

Figure S11 ^{13}C NMR (CDCl$_3$, 25°C) spectrum of **L6**

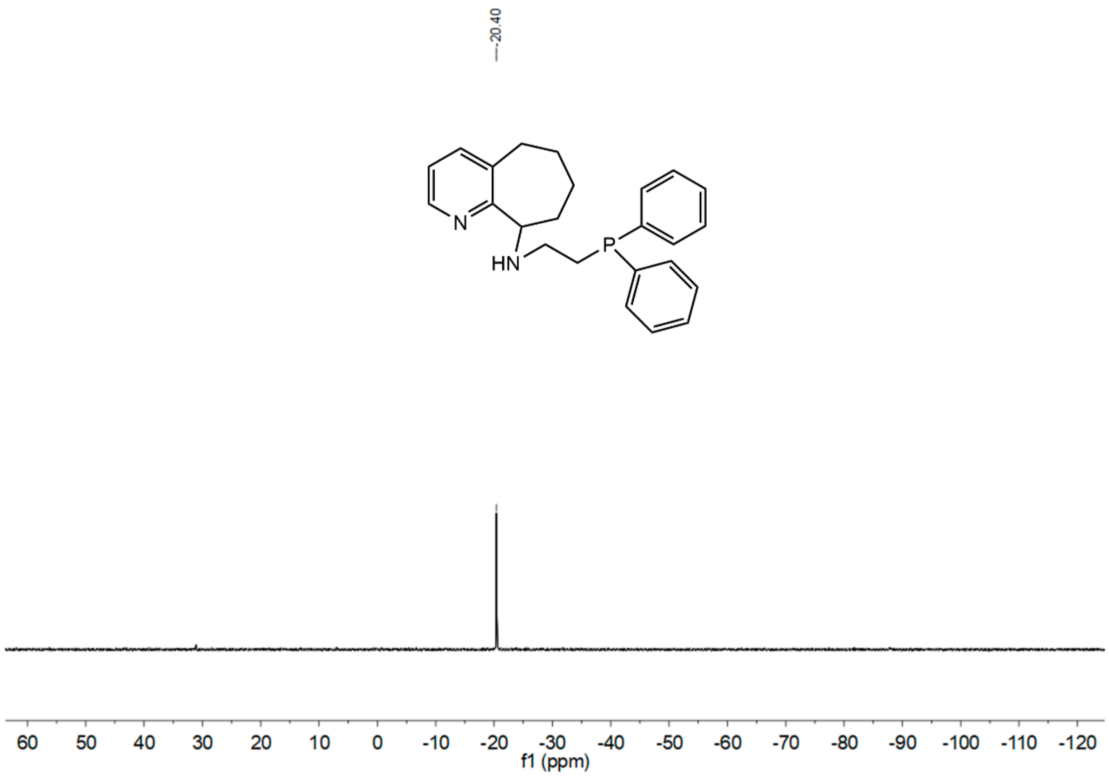

Figure S12 ^{31}P NMR (CDCl$_3$, 25°C) spectrum of **L6**

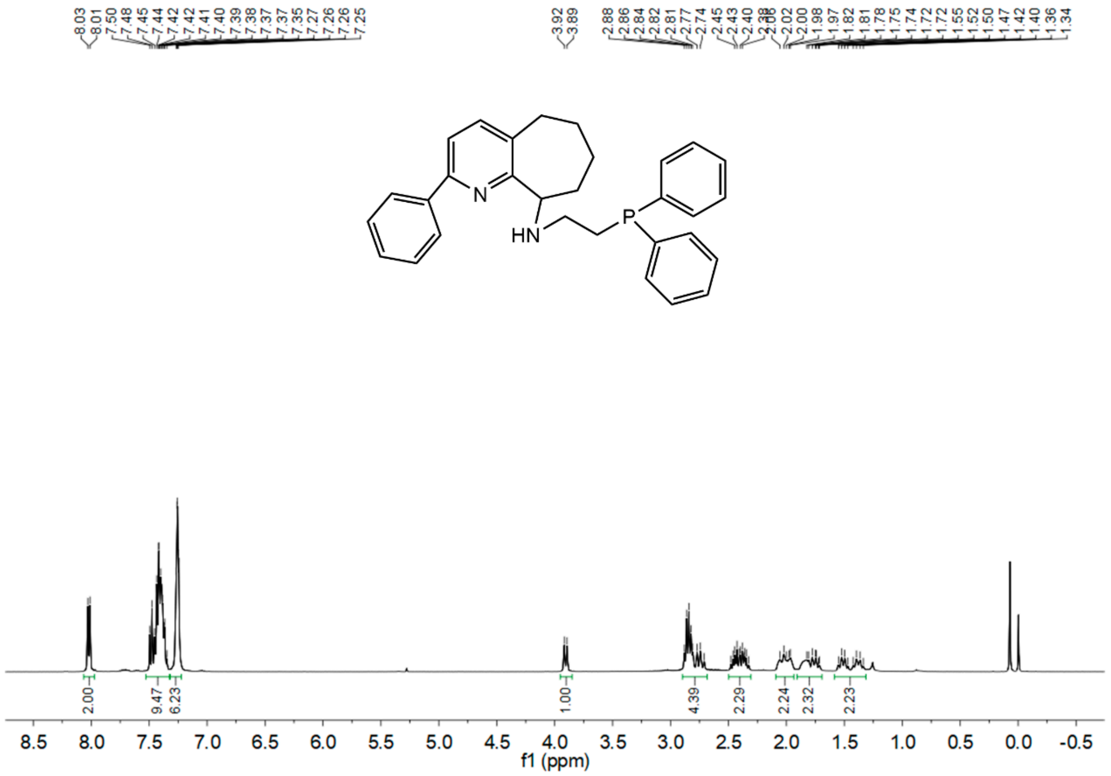

Figure S13 ^{1}H NMR ($CDCl_3$, 25°C) spectrum of **L7**

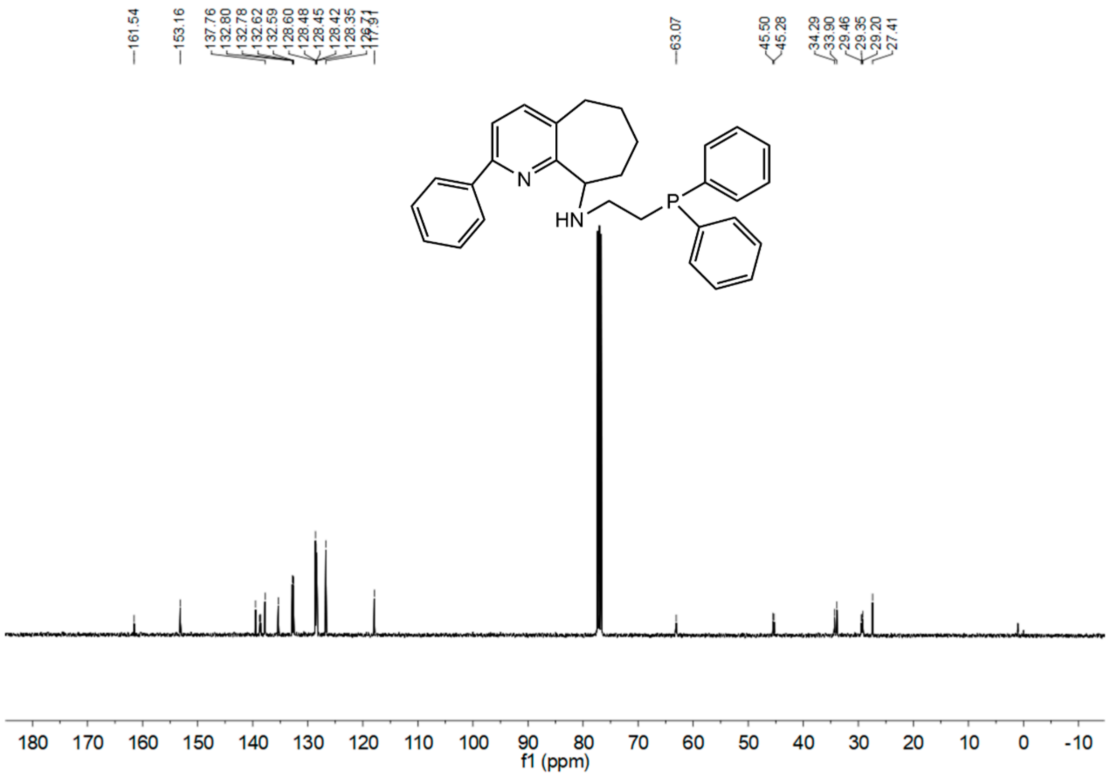

Figure S14 ^{13}C NMR ($CDCl_3$, 25°C) spectrum of **L7**

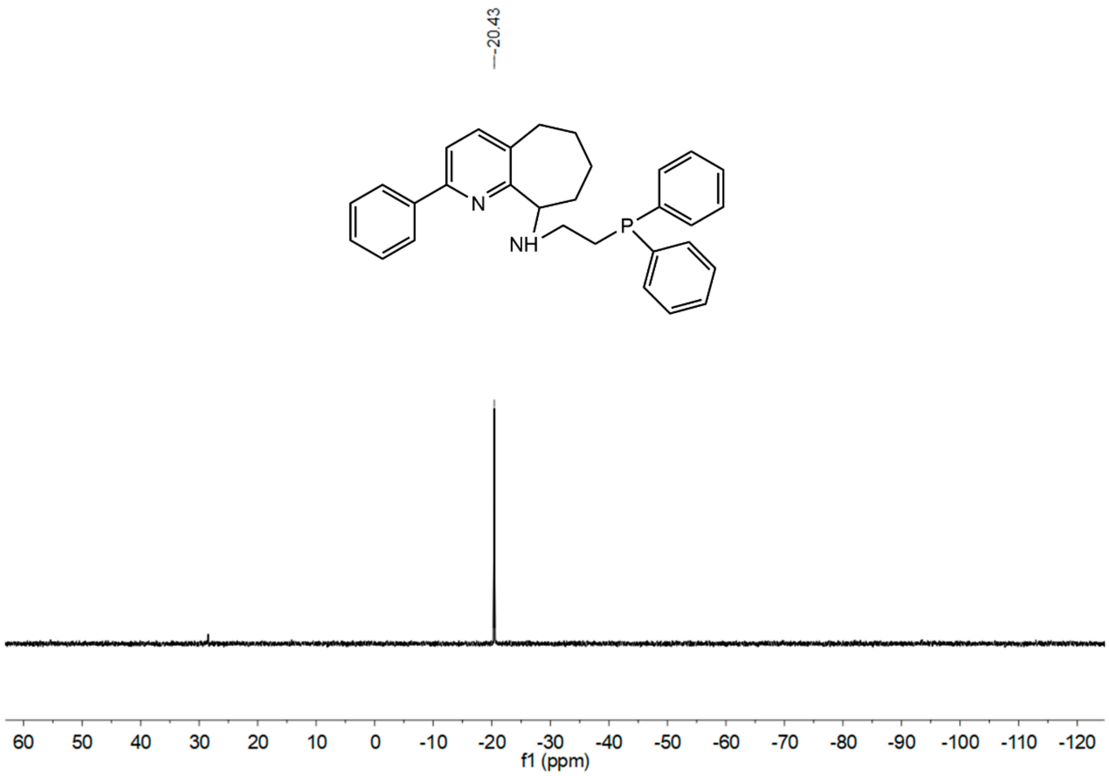

Figure S15 ^{31}P NMR (DMF, 25°C) spectrum of **L7**

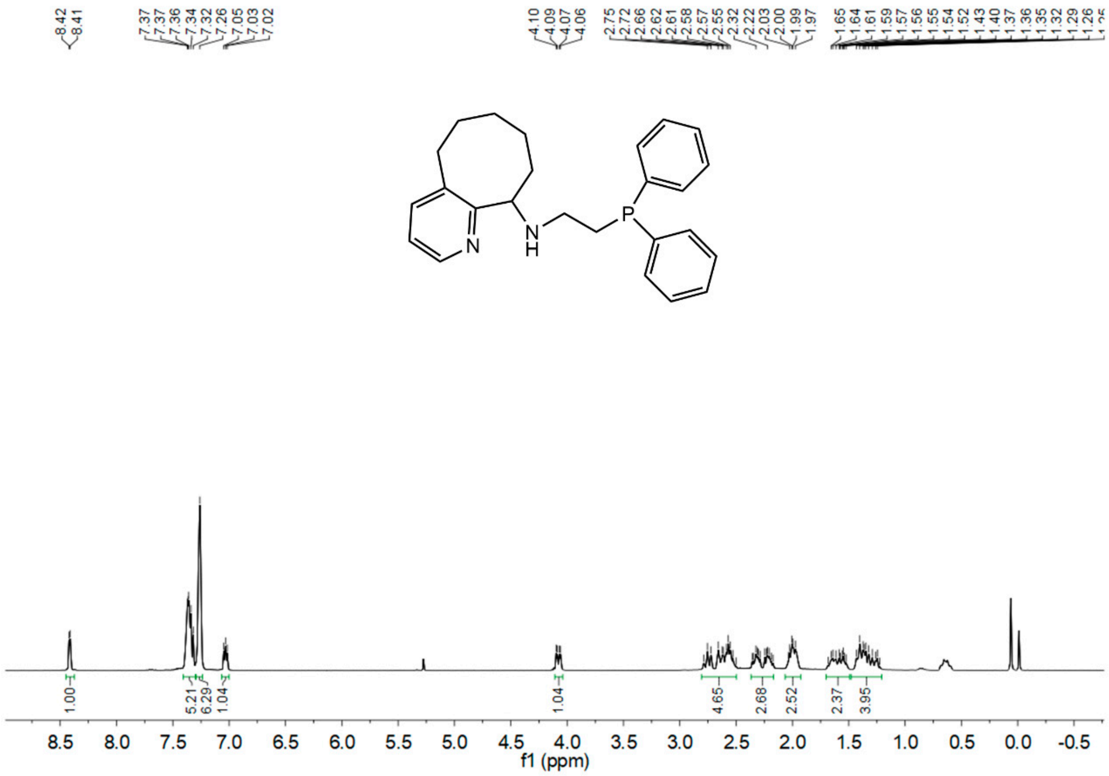

Figure S16 ^{1}H NMR ($CDCl_3$, 25°C) spectrum of **L8**

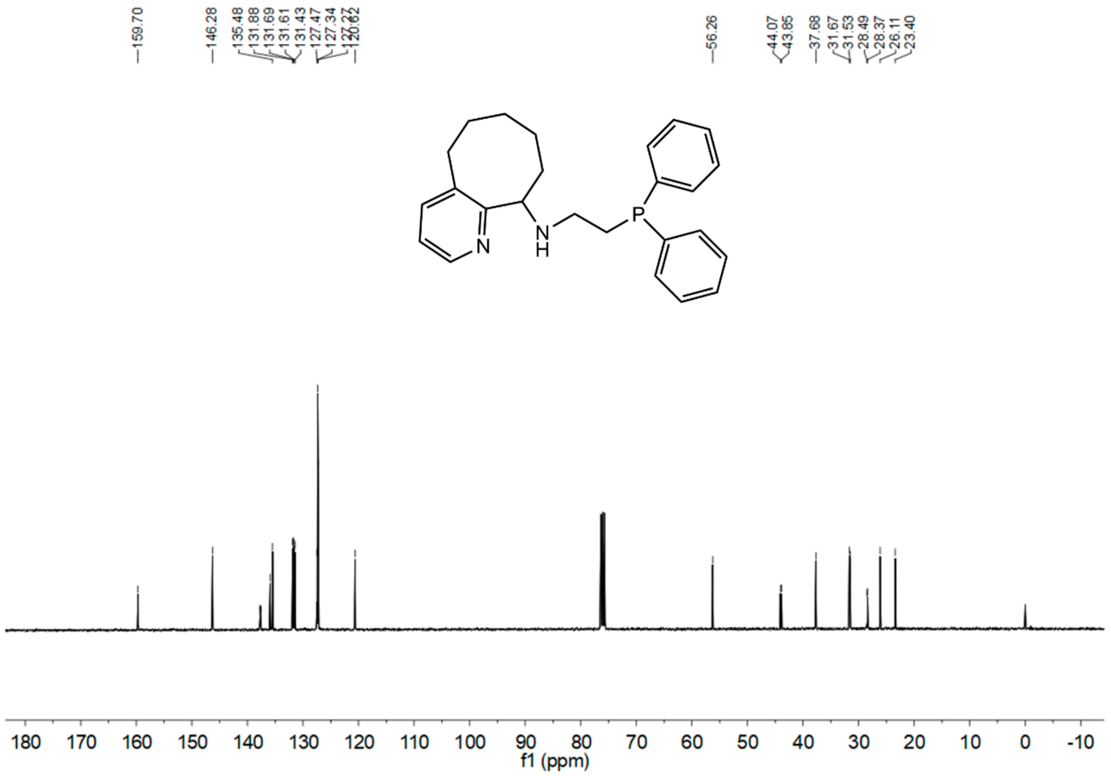

Figure S17 ^{13}C NMR ($CDCl_3$, 25°C) spectrum of **L8**

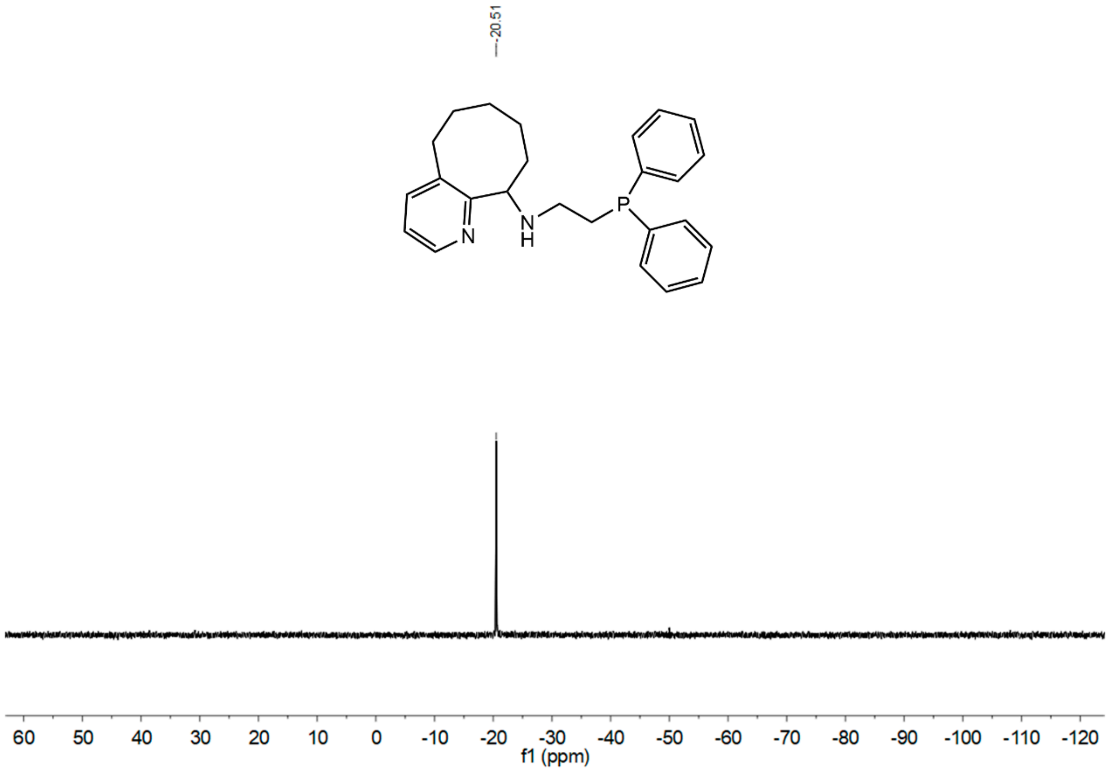

Figure S18 ^{31}P NMR ($CDCl_3$, 25°C) spectrum of **L8**

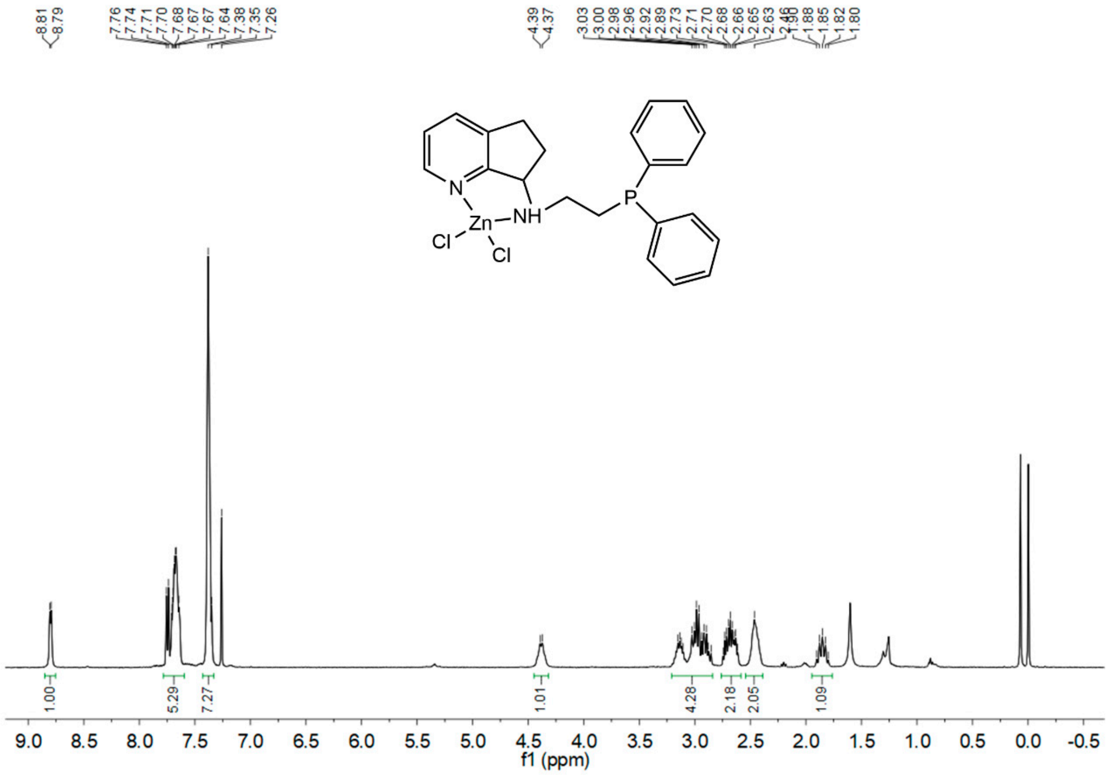

Figure S19 ^{1}H NMR ($CDCl_3$, 25°C) spectrum of **Zn1**

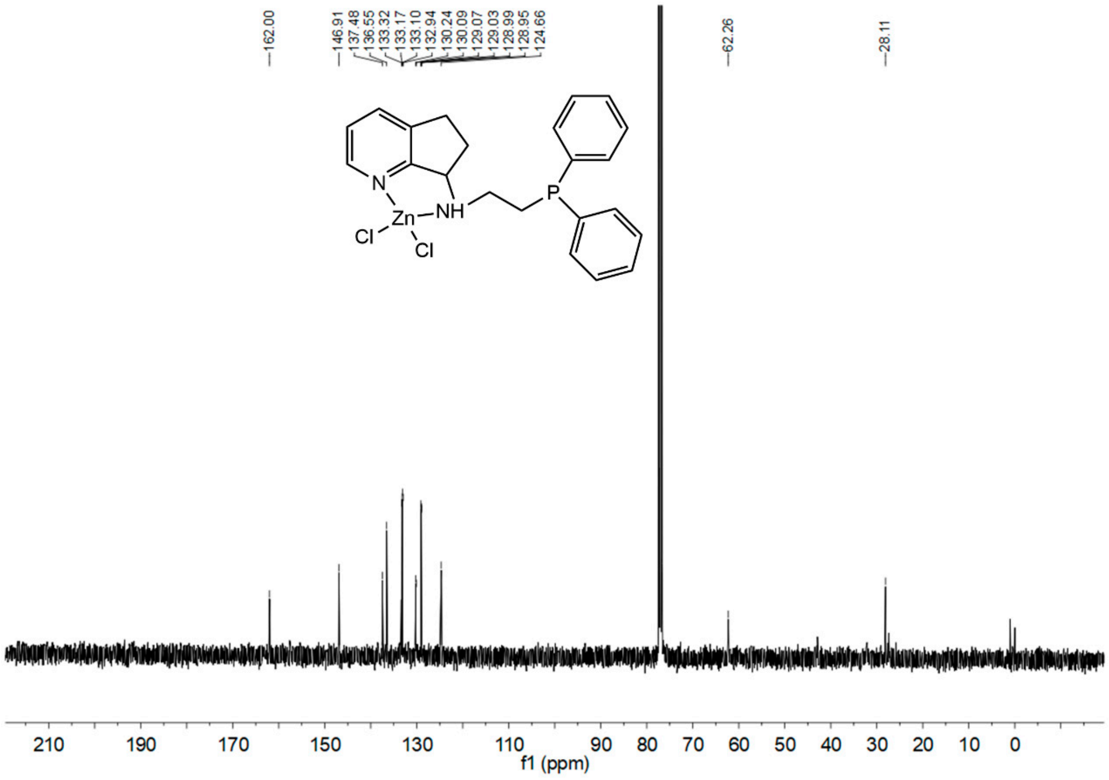

Figure S20 ^{13}C NMR ($CDCl_3$, 25°C) spectrum of **Zn1**

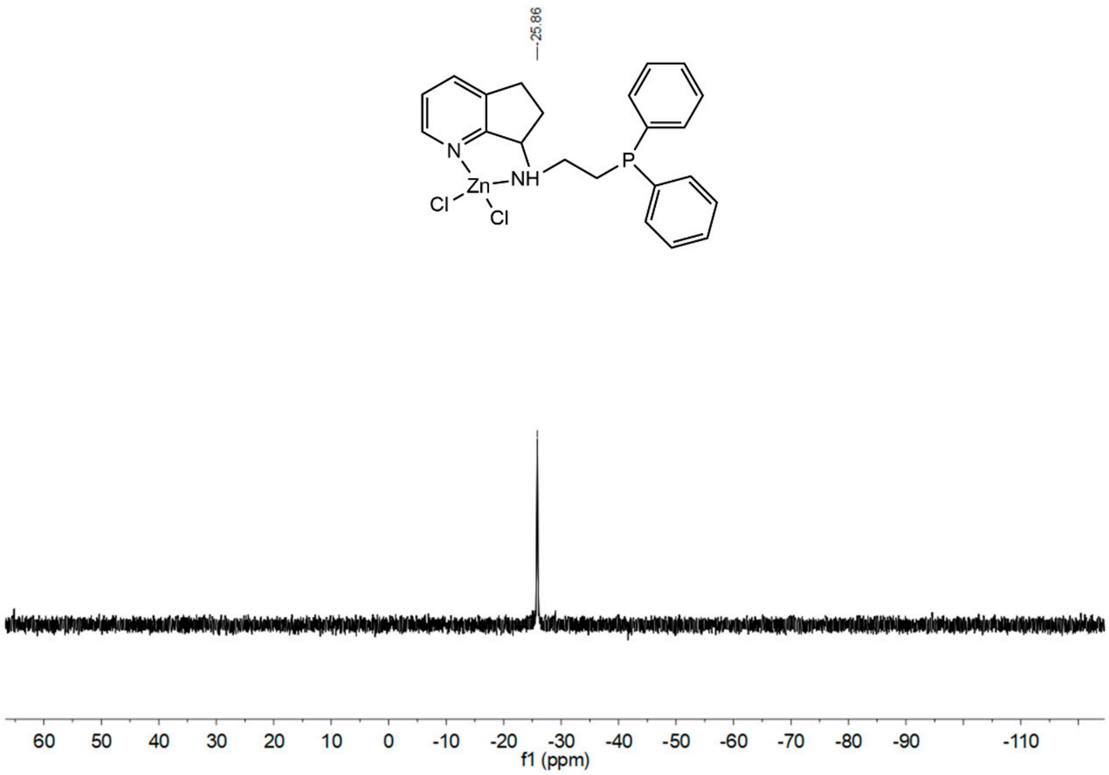

Figure S21 ^{31}P NMR ($CDCl_3$, 25°C) spectrum of **Zn1**

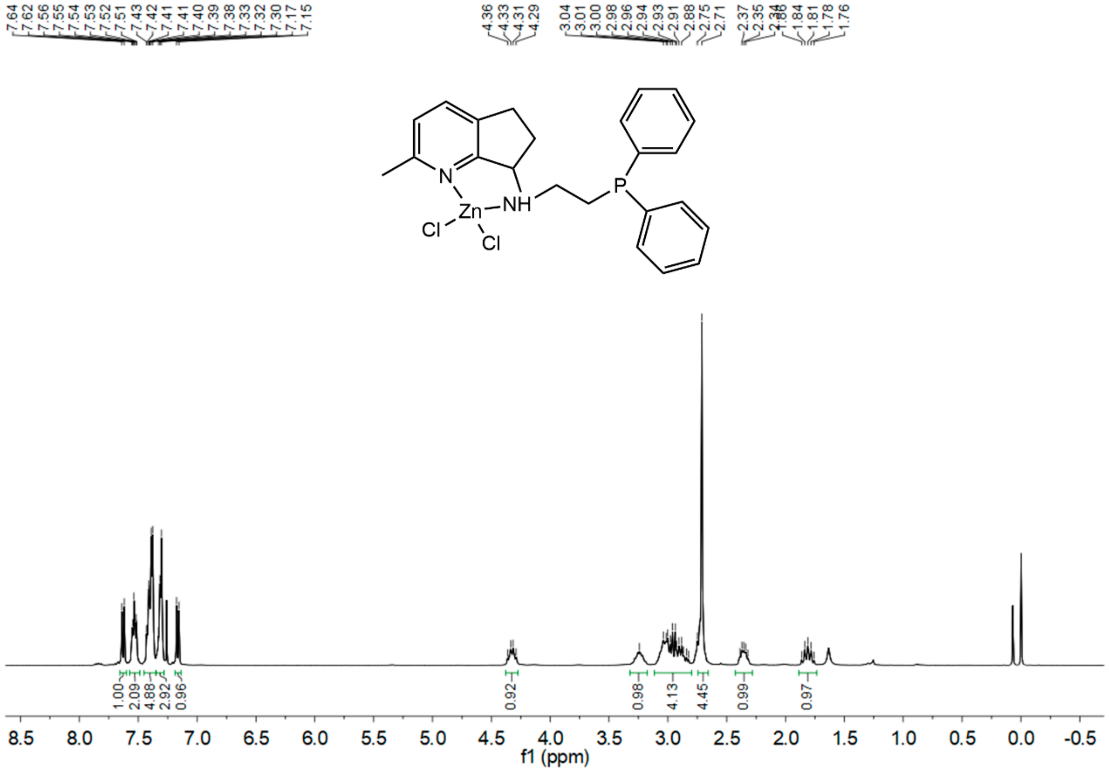

Figure S22 ^{1}H NMR ($CDCl_3$, 25°C) spectrum of **Zn2**

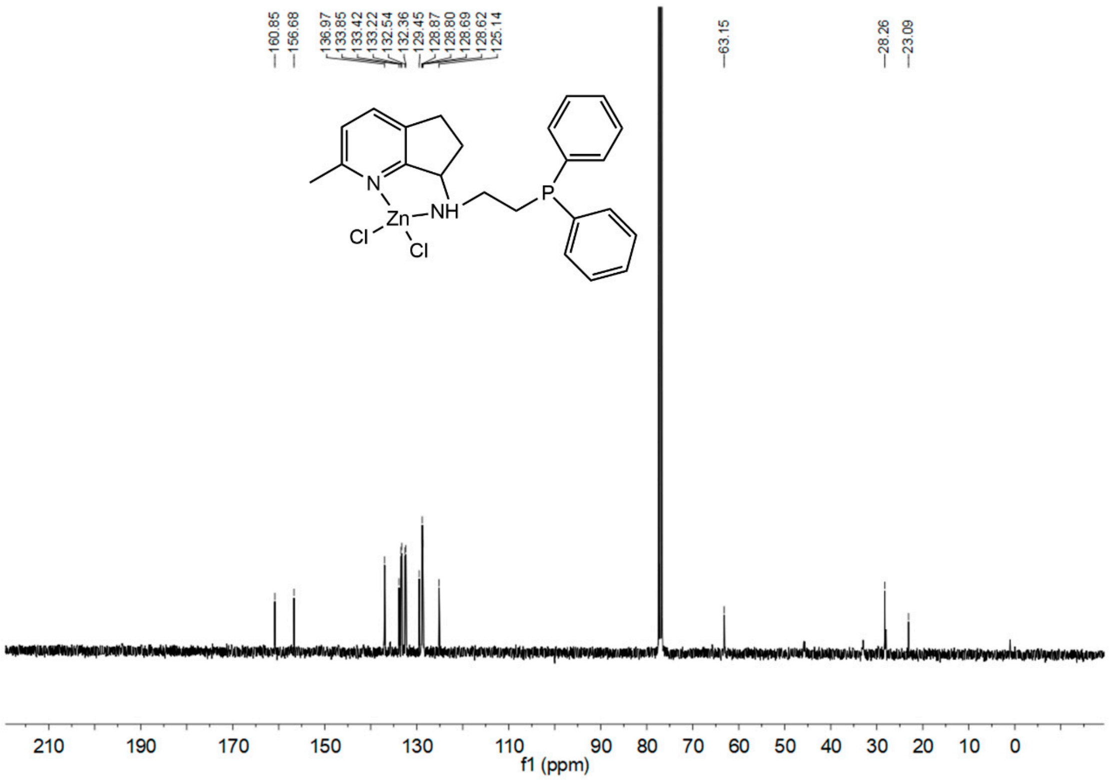

Figure S23 ^{13}C NMR ($CDCl_3$, 25°C) spectrum of **Zn2**

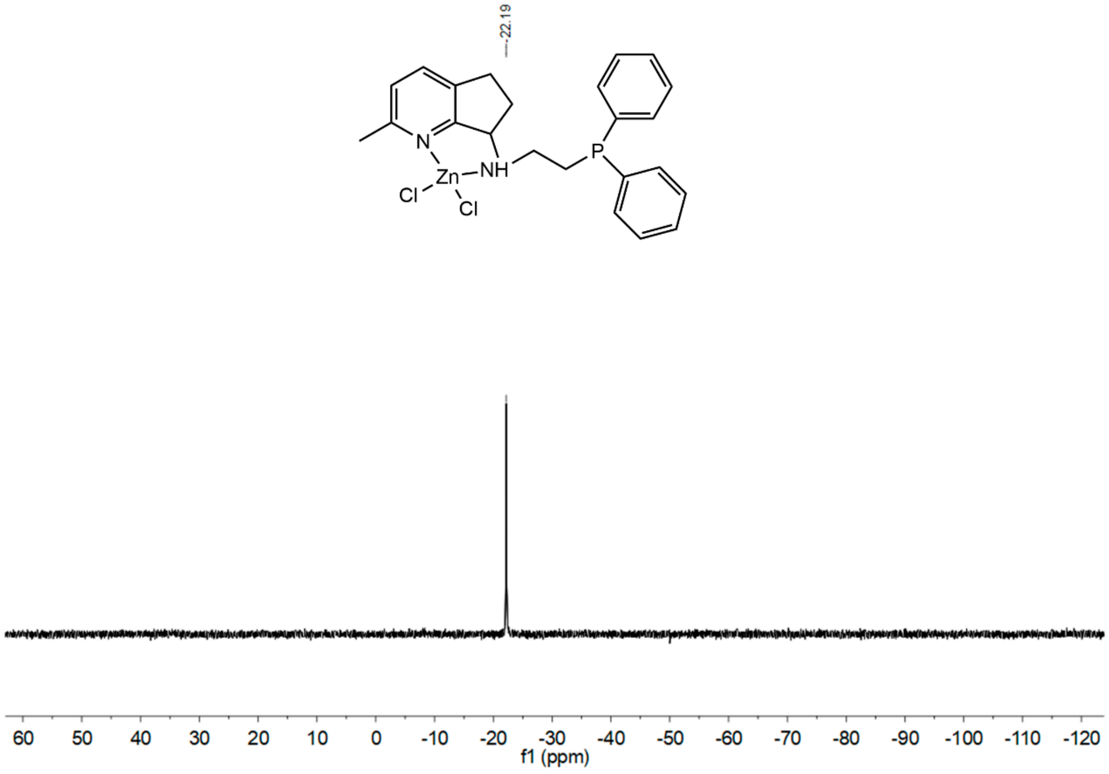

Figure S24 ^{31}P NMR ($CDCl_3$, 25°C) spectrum of **Zn2**

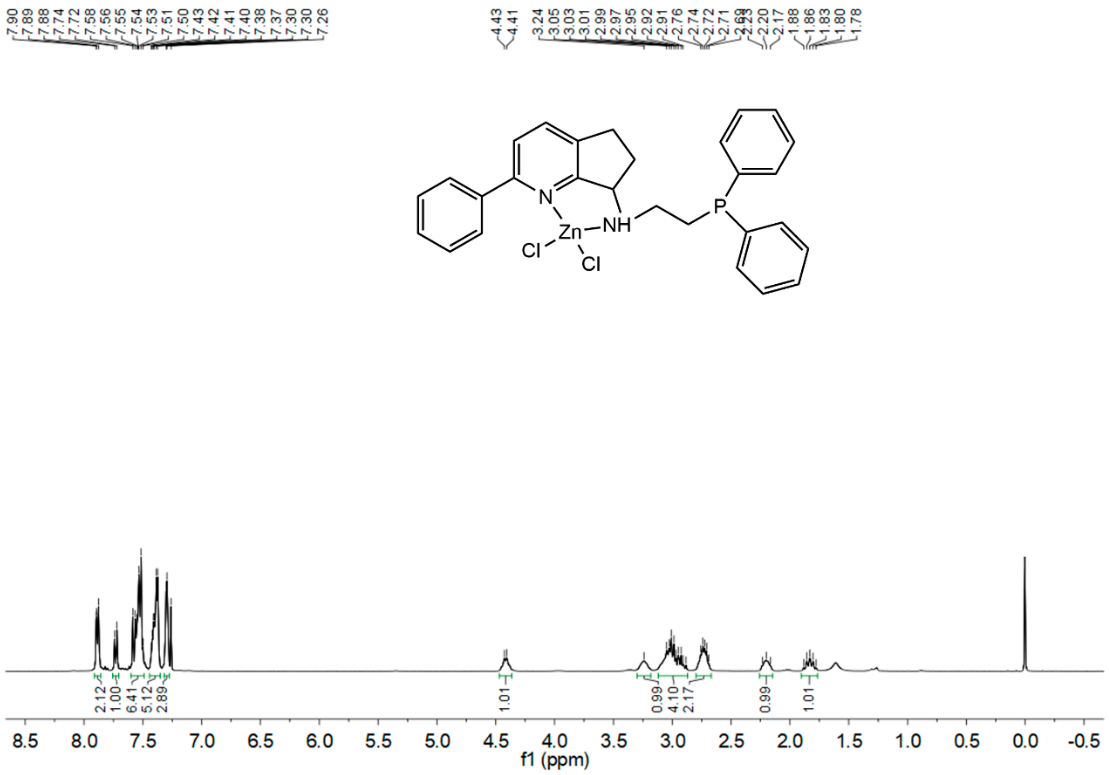

Figure S25 ^{1}H NMR ($CDCl_3$, 25°C) spectrum of **Zn3**

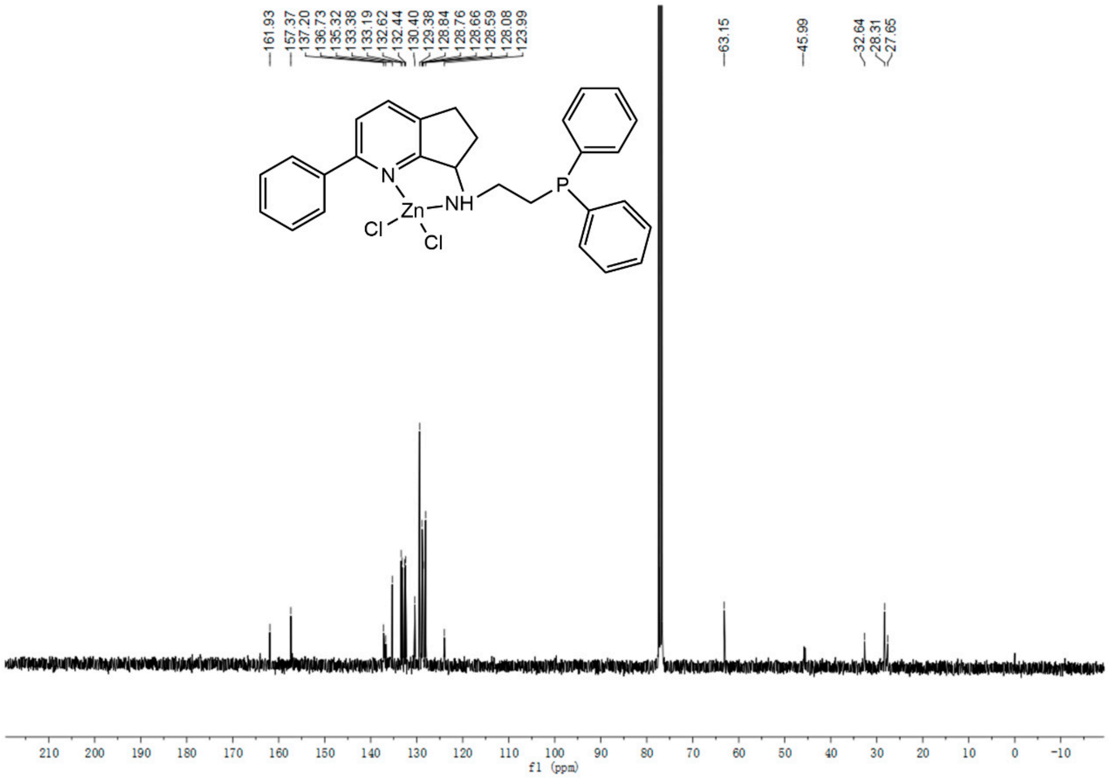

Figure S26 ^{13}C NMR ($CDCl_3$, 25°C) spectrum of **Zn3**

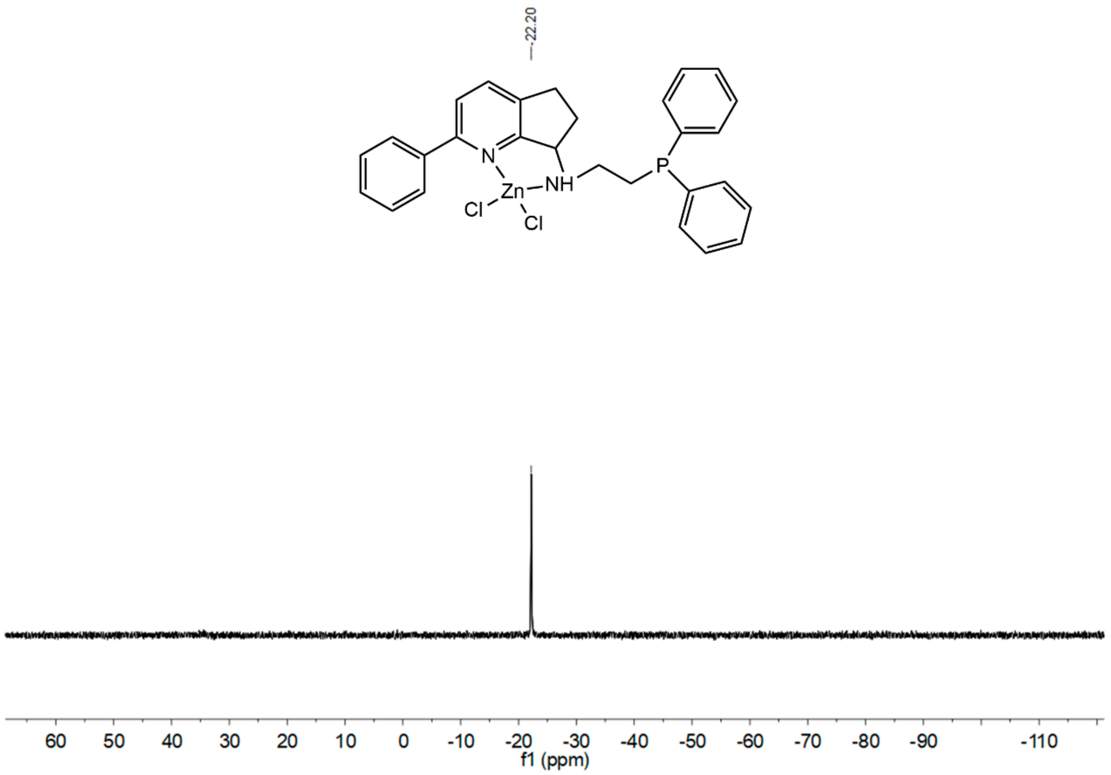

Figure S27 ^{31}P NMR ($CDCl_3$, 25°C) spectrum of **Zn3**

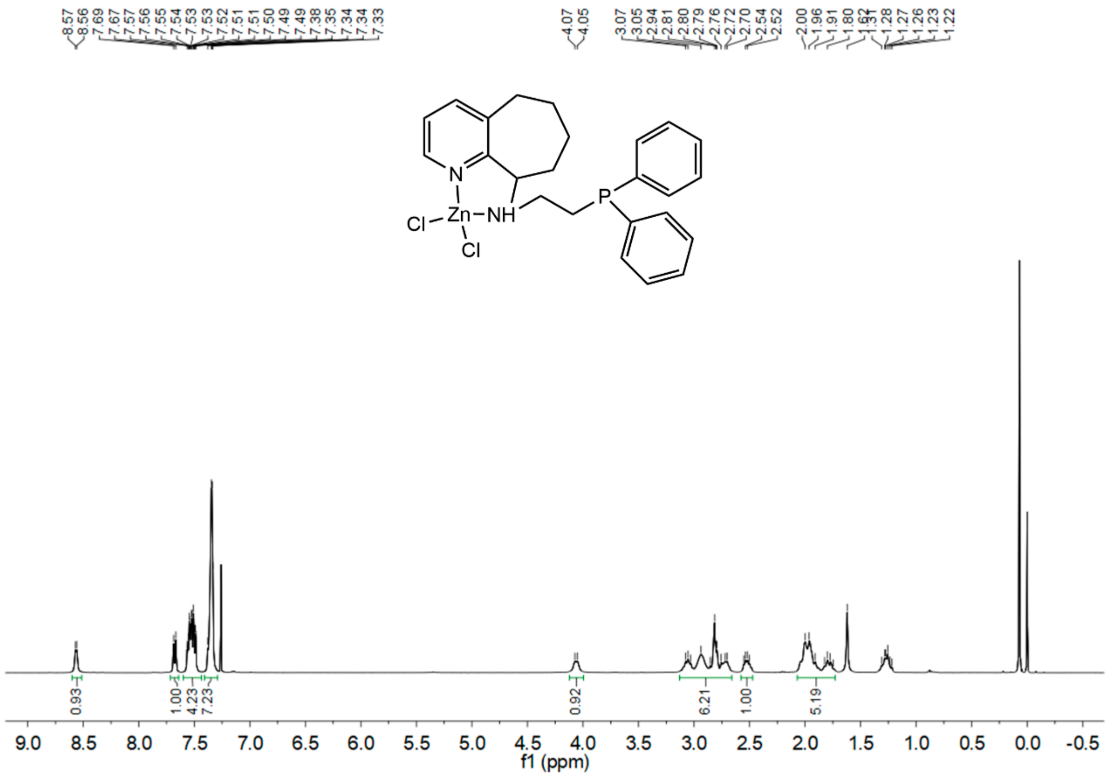

Figure S28 ^{1}H NMR ($CDCl_3$, 25°C) spectrum of **Zn6**

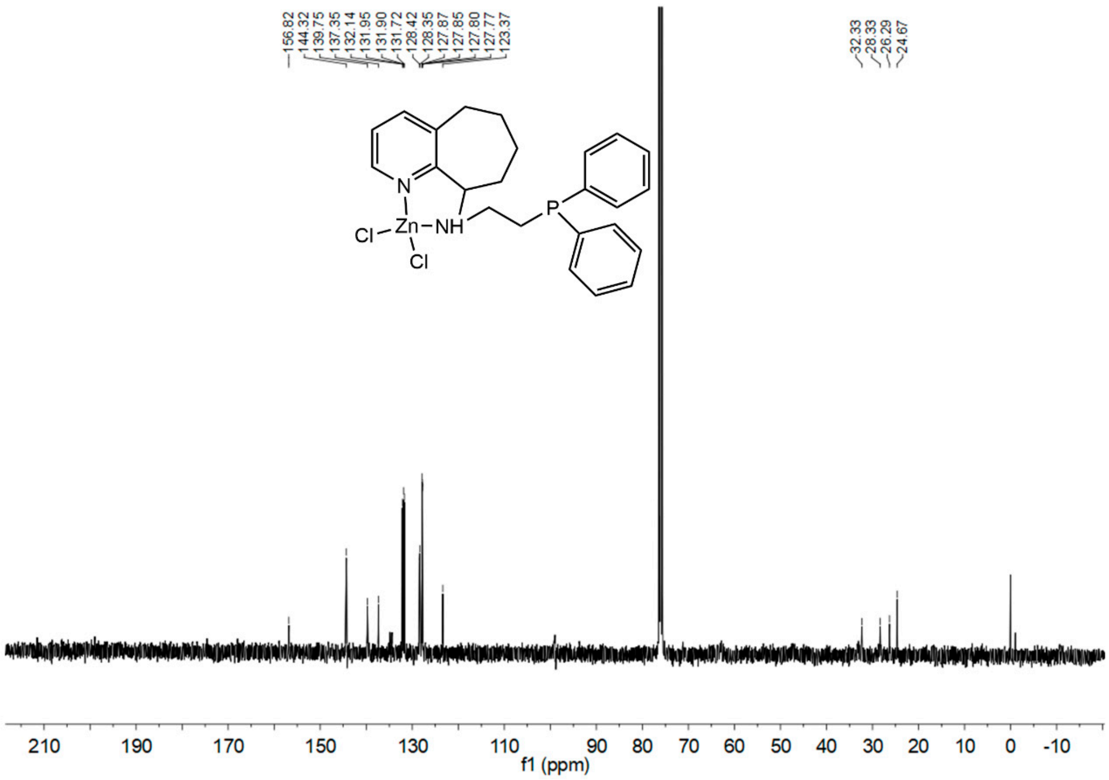

Figure S29 ^{13}C NMR ($CDCl_3$, 25°C) spectrum of **Zn6**

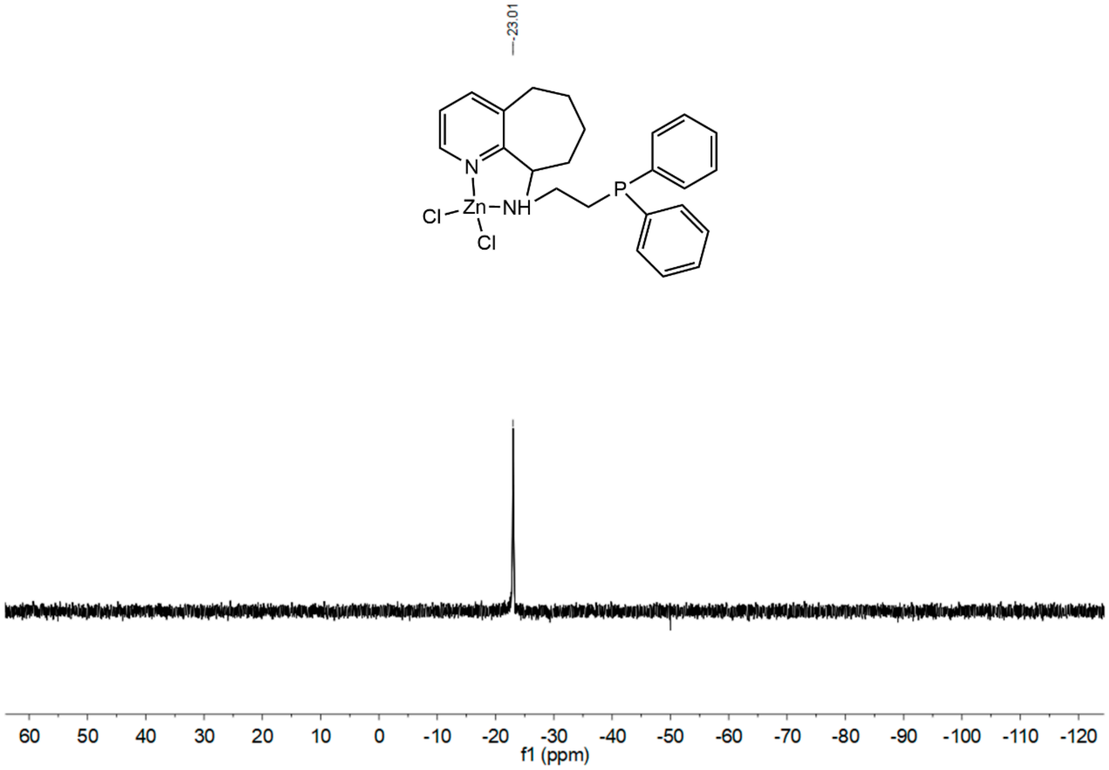

Figure S30 ^{31}P NMR ($CDCl_3$, 25°C) spectrum of **Zn6**

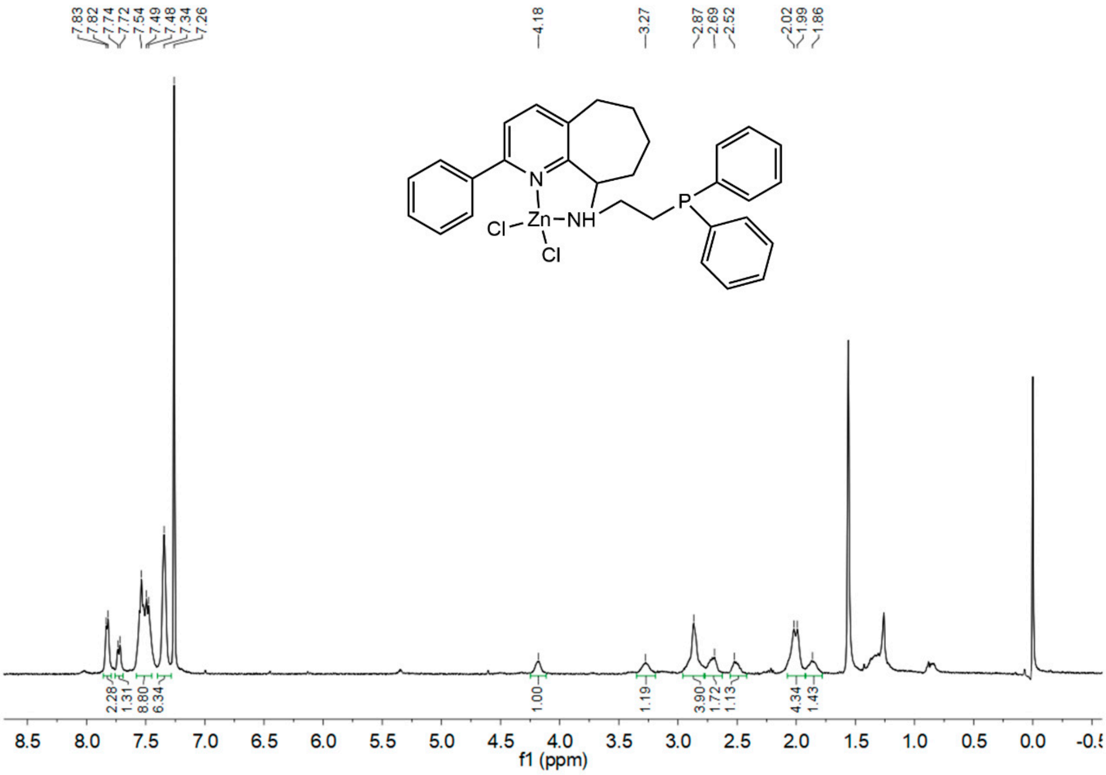

Figure S31 ^{1}H NMR ($CDCl_3$, 25°C) spectrum of **Zn7**

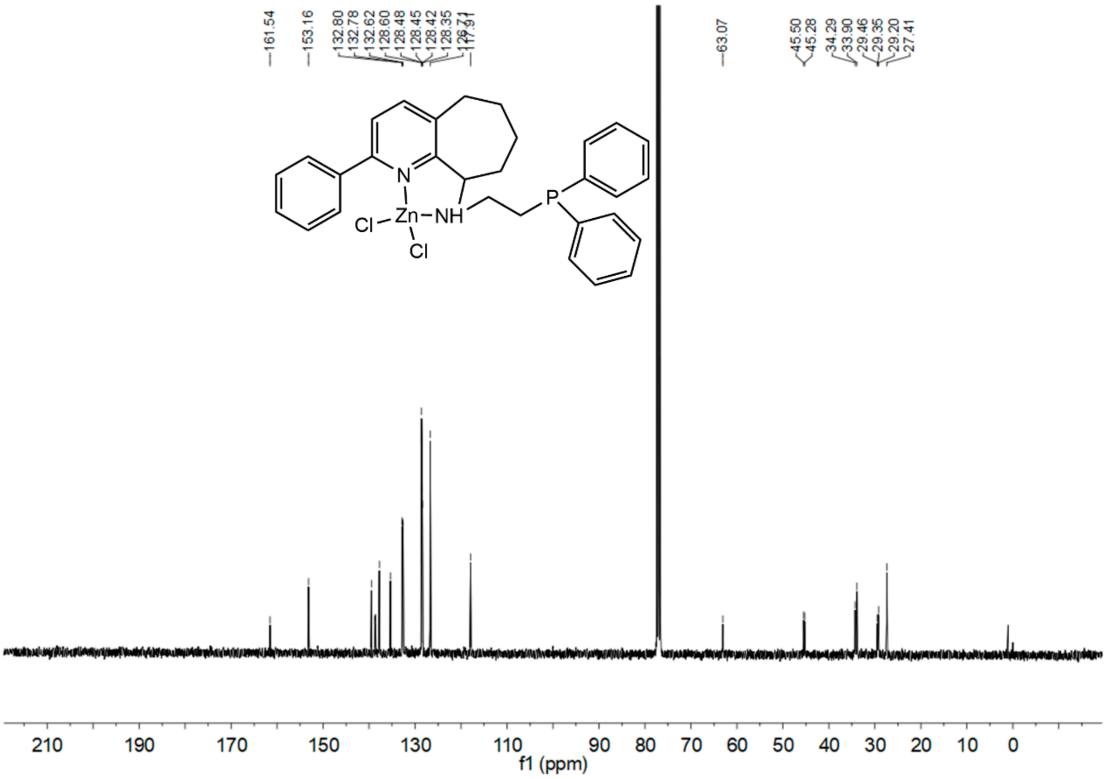

Figure S32 ^{13}C NMR ($CDCl_3$, 25°C) spectrum of **Zn7**

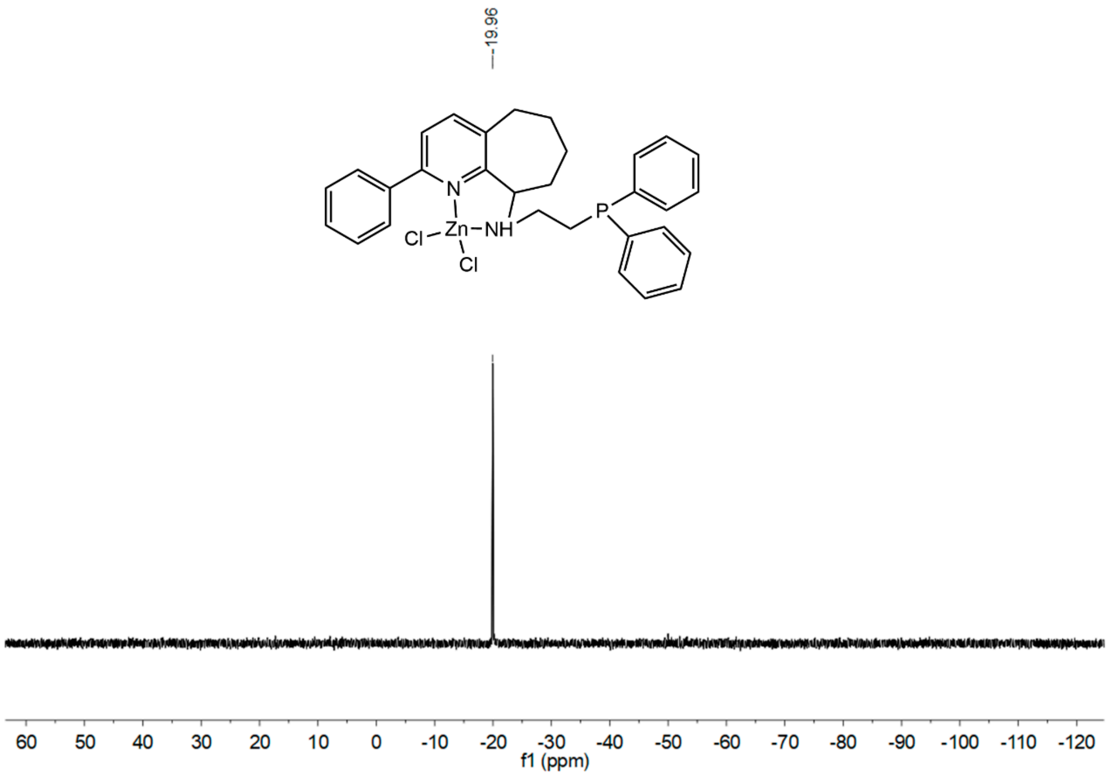

Figure S33 ^{31}P NMR (DMF, 25°C) spectrum of **Zn7**

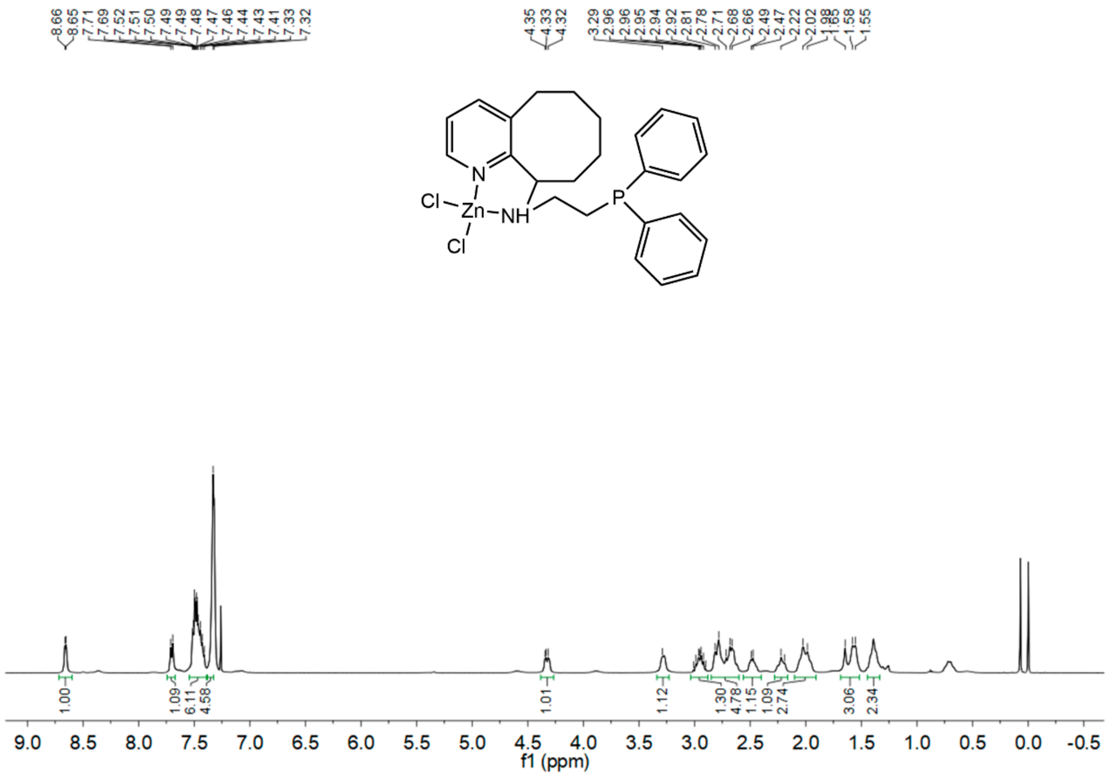

Figure S34 ^{1}H NMR ($CDCl_3$, 25°C) spectrum of **Zn8**

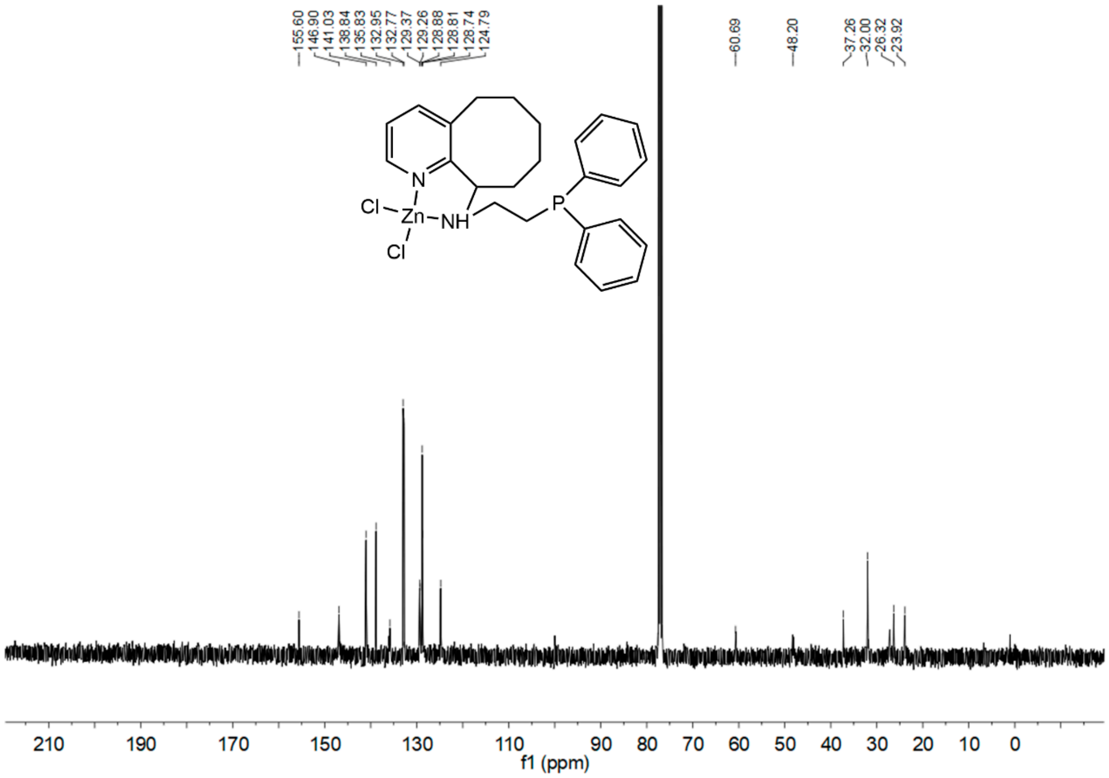

Figure S35 ^{13}C NMR ($CDCl_3$, 25°C) spectrum of **Zn8**

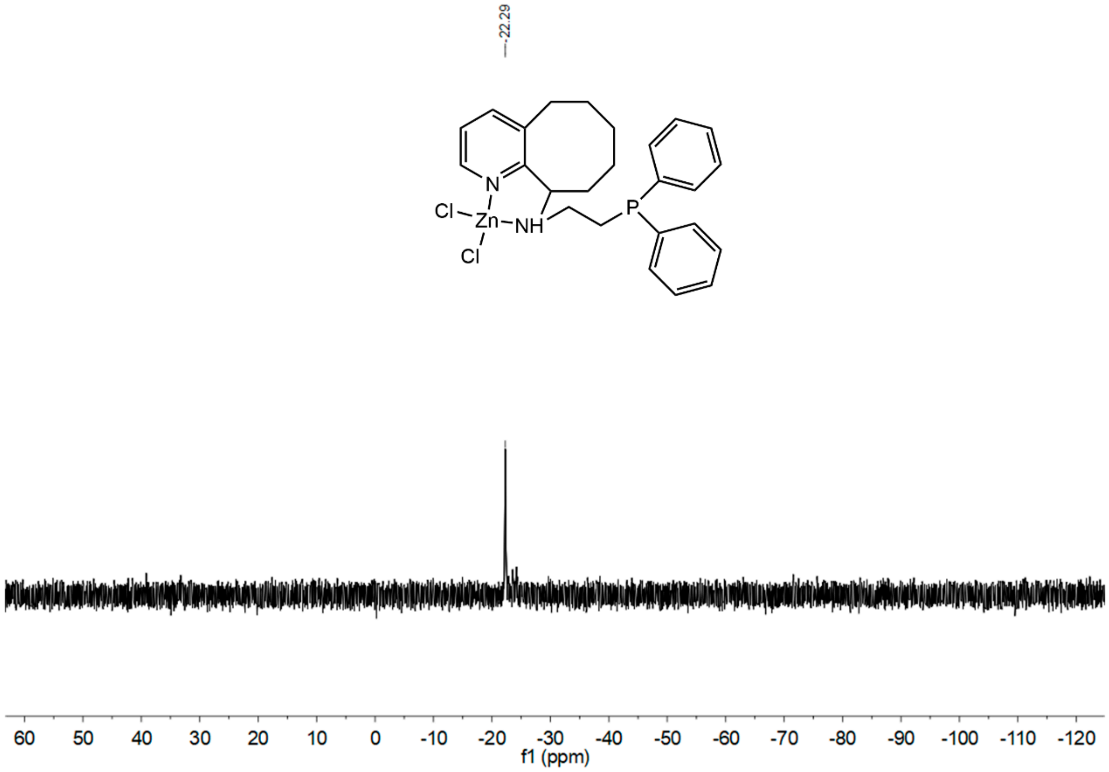

Figure S36 ^{31}P NMR ($CDCl_3$, 25°C) spectrum of **Zn8**

Table S1 Comparison of the ^{31}P NMR chemical shifts for ligands with those in zinc complexes

Ligands	^{31}P NMR chemical shift (ppm)	Complex	^{31}P NMR chemical shift (ppm)
**L1** (H)	-20.74	**Zn1**	-25.86
**L2** (Me)	-18.57	**Zn2**	-22.19
**L3** (Ph)	-20.35	**Zn3**	-22.20
**L6** (H)	-20.40	**Zn6**	-23.01
**L7** (Ph)	-20.43 (DMF)	**Zn7**	-19.96 (DMF)
**L8** (H)	-20.51	**Zn8**	-22.29

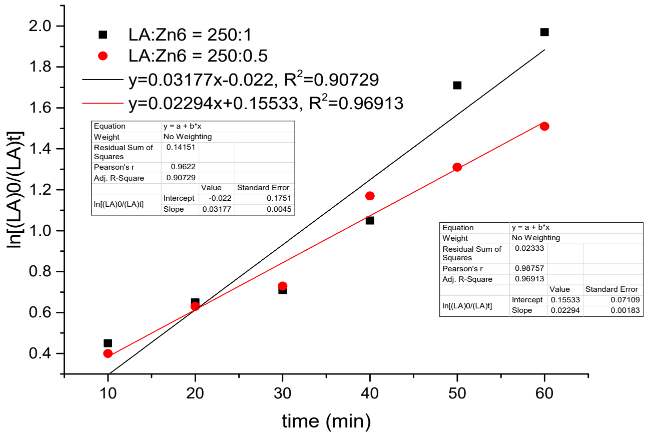

Figure S37 Kinetics plots of polymerization of *rac*-LA by **Zn6**/2LiN$(SiMe_3)_2$ (LA:Zn = 250:1 or 250:0.5, 30°C, toluene)

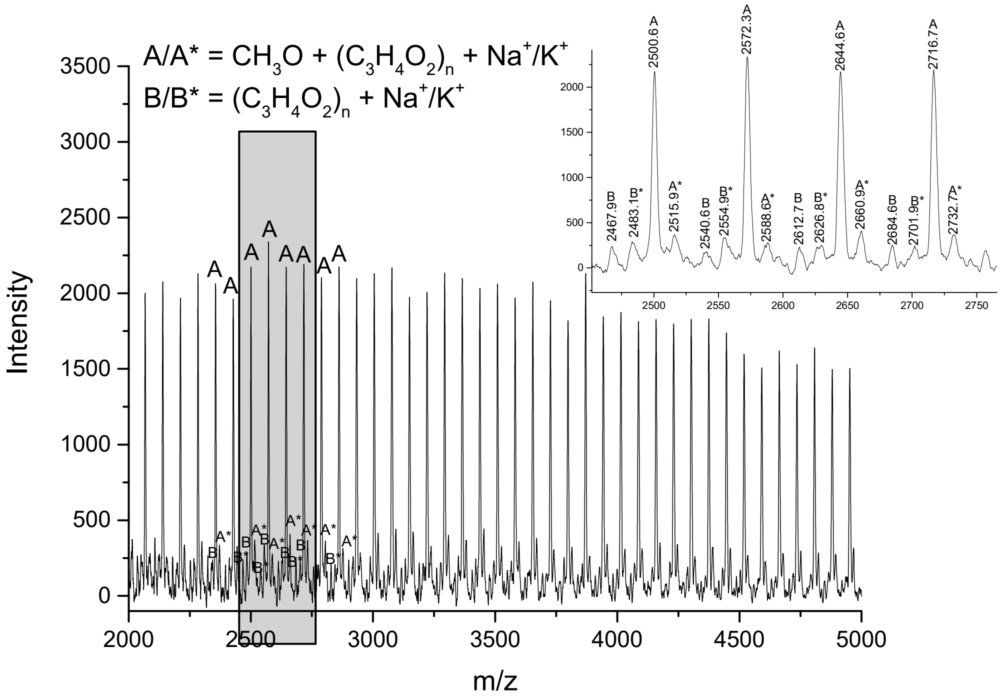

Figure S38 MALDI-TOF spectrum of the poly(*rac*-LA) obtained using **Zn6**/2LiN(SiMe3)2 (run 1, Table 3)

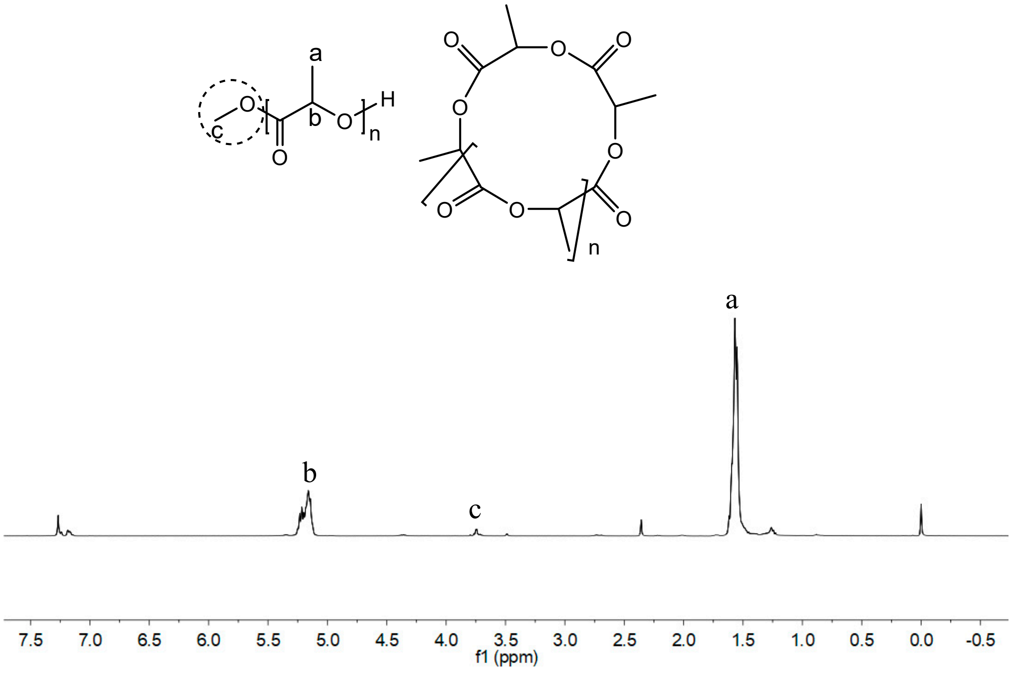

Figure S39 ^{1}H NMR spectrum of the poly(*rac*-LA) obtained using **Zn6**/2LiN(SiMe3)2 (run 1, Table 3)

Peak	Integration	Pm
rmr	0.23	0.33
rmm	0.12	0.4
mmr	0.11	0.32
mmm	0.22	0.33
mrm	0.32	0.36
	Average	0.35

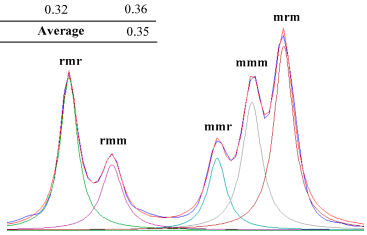

Zn1

Peak	Integration	Pm
rmr	0.24	0.31
rmm	0.11	0.34
mmr	0.11	0.34
mmm	0.23	0.34
mrm	0.30	0.4
	Average	0.34

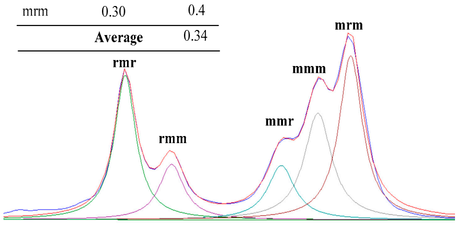

Zn2

Peak	Integration	Pm
rmr	0.25	0.30
rmm	0.10	0.28
mmr	0.10	0.28
mmm	0.20	0.30
mrm	0.34	0.32
	Average	0.30

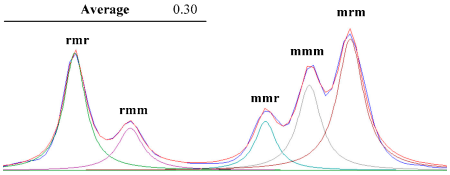

Zn3

Peak	Integration	Pm
rmr	0.25	0.30
rmm	0.11	0.32
mmr	0.10	0.28
mmm	0.21	0.31
mrm	0.33	0.34
	Average	0.31

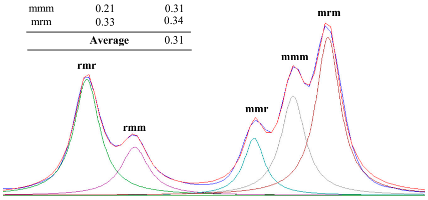

Zn5

Peak	Integration	Pm
rmr	0.23	0.33
rmm	0.12	0.40
mmr	0.10	0.28
mmm	0.22	0.33
mrm	0.32	0.34
	Average	0.33

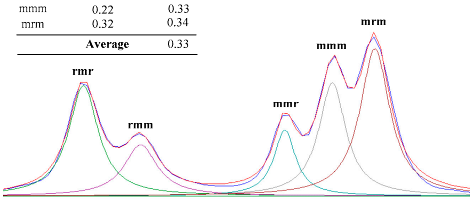

Zn6

Peak	Integration	Pm
rmr	0.23	0.33
rmm	0.12	0.40
mmr	0.11	0.32
mmm	0.23	0.34
mrm	0.31	0.38
	Average	0.35

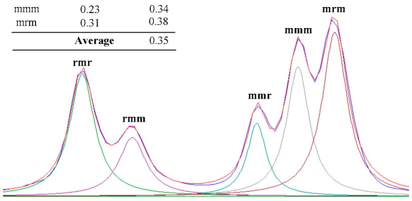

Zn8

Figure S40 decoupling ^{1}H NMR spectrum of the poly(*rac*-LA) obtained using **Zn1-Zn8**/2LiN($SiMe_3$)$_2$ (Table 3)
